# SRPK1 maintains acute myeloid leukemia through effects on isoform usage of epigenetic regulators including BRD4

**DOI:** 10.1038/s41467-018-07620-0

**Published:** 2018-12-19

**Authors:** Konstantinos Tzelepis, Etienne De Braekeleer, Demetrios Aspris, Isaia Barbieri, M. S. Vijayabaskar, Wen-Hsin Liu, Malgorzata Gozdecka, Emmanouil Metzakopian, Hamish D. Toop, Monika Dudek, Samuel C. Robson, Francisco Hermida-Prado, Yu Hsuen Yang, Roya Babaei-Jadidi, Dimitrios A. Garyfallos, Hannes Ponstingl, Joao M. L. Dias, Paolo Gallipoli, Michael Seiler, Silvia Buonamici, Binje Vick, Andrew J. Bannister, Roland Rad, Rab K. Prinjha, John C. Marioni, Brian Huntly, Jennifer Batson, Jonathan C. Morris, Cristina Pina, Allan Bradley, Irmela Jeremias, David O. Bates, Kosuke Yusa, Tony Kouzarides, George S. Vassiliou

**Affiliations:** 1 Haematological Cancer Genetics, Wellcome Trust Sanger Institute, Hinxton, Cambridge, CB10 1SA UK; 2 Gurdon Institute and Department of Pathology, Tennis Court Road, Cambridge, CB2 1QN UK; 3 Karaiskakio Foundation, Nicosia, Cyprus; 4 Division of Cellular and Molecular Pathology, Department of Pathology, University of Cambridge, Addenbrookes Hospital, CB2 0QQ Cambridge, UK; 5 Research Unit Apoptosis in Hematopoietic Stem Cells, Helmholtz Zentrum München, German Research Center for Environmental Health (HMGU), 81377 Munich, Germany; 6 Wellcome Trust–MRC Cambridge Stem Cell Institute, University of Cambridge, Cambridge, CB2 0XY UK; 7 UK Dementia Research Institute, University of Cambridge, Hills Rd, Cambridge, CB2 0AH UK; 8 School of Chemistry, University of New South Wales, Sydney, Australia; 9 Exonate Ltd, Milton Science Park, Cambridge, UK; 10 School of Pharmacy and Biomedical Science, University of Portsmouth, White Swan Road, Portsmouth, PO1 2DT UK; 11 Wellcome Trust Sanger Institute, Genome Campus, Hinxton, Cambridge, CB10 1SA UK; 12 Cancer Molecular Diagnosis Laboratory, National Institute for Health Research, Biomedical Research Centre, University of Cambridge, Cambridge, UK; 13 Department of Haematology, University of Cambridge, Cambridge, CB2 0PT UK; 14 Department of Haematology, Cambridge University Hospitals NHS Trust, Cambridge, CB2 0QQ UK; 15 H3 Biomedicine Inc., Cambridge, MA USA; 16 Institute of Molecular Oncology and Functional Genomics, Department of Medicine II and TranslaTUM Cancer Center, Technical University of Munich, Germany; 17 German Cancer Research Center (DKFZ), Heidelberg, & German Cancer Consortium (DKTK), Heidelberg, Germany; 18 Epigenetics DPU, Immunoinflammation and Oncology TA Unit, GSK Medicines Research Centre, Gunnels Wood Road, Stevenage, SG1 2NY UK; 19 Cancer Research UK Cambridge Institute, University of Cambridge, Robinson Way, Cambridge, CB2 0RE UK; 20 European Bioinformatics Institute, Wellcome Genome Campus, Hinxton, Cambridgeshire, CB10 1SD UK; 21 Stem Cell Genetics, Wellcome Trust Sanger Institute, Hinxton, Cambridge, CB10 1SA UK; 22 Department of Pediatrics, Dr. von Hauner Children’s Hospital, Ludwig Maximilians University München, 80337 Munich, Germany; 23 Cancer Biology, Division of Cancer and Stem Cells, School of Medicine, University of Nottingham, Queen′s Medical Centre, Nottingham, NG2 7UH UK

## Abstract

We recently identified the splicing kinase gene *SRPK1* as a genetic vulnerability of acute myeloid leukemia (AML). Here, we show that genetic or pharmacological inhibition of SRPK1 leads to cell cycle arrest, leukemic cell differentiation and prolonged survival of mice transplanted with *MLL*-rearranged AML. RNA-seq analysis demonstrates that SRPK1 inhibition leads to altered isoform levels of many genes including several with established roles in leukemogenesis such as *MYB*, *BRD4* and *MED24*. We focus on *BRD4* as its main isoforms have distinct molecular properties and find that SRPK1 inhibition produces a significant switch from the short to the long isoform at the mRNA and protein levels. This was associated with BRD4 eviction from genomic loci involved in leukemogenesis including *BCL2* and *MYC*. We go on to show that this switch mediates at least part of the anti-leukemic effects of SRPK1 inhibition. Our findings reveal that SRPK1 represents a plausible new therapeutic target against AML.

## Abstract (web summary)

SRPK1, a kinase involved in splicing regulation, is a potential therapeutic target for AML patients. Here, the authors show that SRPK1 inhibition changes isoform levels of key epigenetic regulators, including BRD4, and it has anti-tumor effects specifically against *MLL*-rearranged AML cells.

## Introduction

Acute myeloid leukemia (AML) is an aggressive cancer of hematopoietic stem cells that remains lethal to most sufferers^1^. To address this unmet clinical need, we recently established a CRISPR-Cas9 platform for the performance of genome-wide recessive screens in mammalian cells and used this to identify genetic vulnerabilities of AML cells^2^. Through this work, we identified the splicing kinase gene *SRPK1* as a genetic vulnerability of AMLs driven by MLL fusion genes^2^. SRPK1 functions coordinately with CLK1, another serine-arginine (SR) protein kinase, to regulate the function of SR splicing proteins including SRSF1 and SRSF2^3^. SRPK1 kinase inhibition is known to produce a switch of VEGF-A splicing away from the predominant pro-angiogenic VEGF_165_a isoform and towards the anti-angiogenic VEGF_165_b isoform^4,5^. This is of therapeutic potential in neovascular eye disease^6^, prostate cancer^5^ and other diseases where VEGF-A plays a role^7^. Also, inhibition of SRPKs was proposed to have anti-leukemic properties^8^.

Here we investigate the molecular basis for the requirement for SRPK1 in *MLL*-rearranged AMLs and explore the therapeutic potential of this finding. We report that inhibition of SRPK1 using gRNA or the specific inhibitor SPHINX31^6^ leads to cell cycle arrest, leukemic cell differentiation, and prolonged survival of immunocompromised mice transplanted with *MLL*-rearranged AML cells. We then show that SRPK1 inhibition affects isoform usage of a number of genes with established roles in leukemogenesis including *MYB*^9^, *BRD4*^10^, and *MED24*^11^. BRD4 has a well-recognized role in AML maintenance and its two main isoforms have distinct molecular properties, at least in certain contexts^12^, yet it is not known if they have different roles in AML. We go on to show that SRPK1 inhibition leads to a marked switch from the short to the long BRD4 isoform at both the mRNA and the protein levels. This was associated with eviction of BRD4 from genomic loci that were previously shown to be required for myeloid leukemogenesis, including *BCL2* and *MYC*. Using rescue experiments we demonstrate that the *BRD4* switch *per se* has anti-leukemic properties. Collectively, our work reveals that SRPK1 inhibition is a plausible therapeutic strategy in AML and gives insights into the molecular basis of this finding.

## Results

### Loss of SRPK1 halts AML expansion in vitro and in vivo

**Fig. 1:**
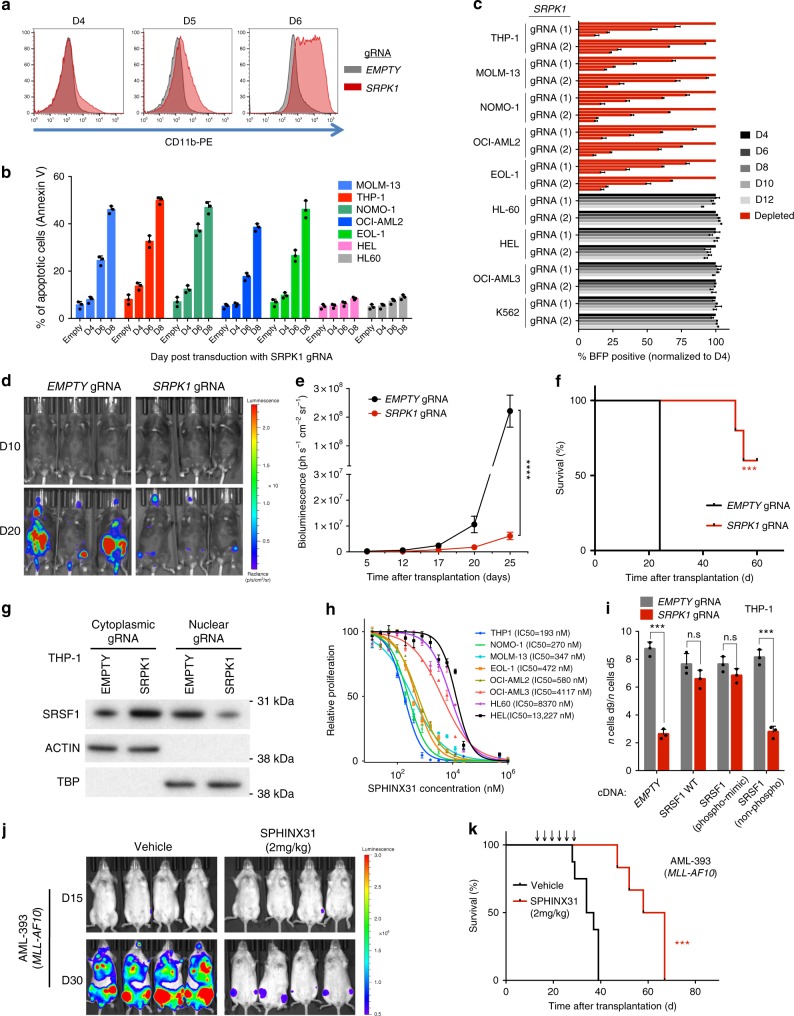
Genetic and pharmacological inhibition of SRPK1 inhibits growth and drives differentiation of human AML cells. **a** CD11b expression in MOLM-13 cells 4, 5, and 6 days after gRNA. **b** Increased apoptosis levels in AML cells driven by MLL-X fusion genes (MOLM13, THP1, NOMO-1, and OCI-AML2) or MLL-PTD (EOL-1) after dual gRNA targeting of *SRPK1* (mean ± s.d., *n* = 3). **c** Competitive co-culture of lentiviral *SRPK1* gRNA-transfected (BFP positive) vs untransfected AML cell lines normalized to %BFP on day 4 (mean ± s.d., *n* = 3). **d** Bioluminescence imaging of mice transplanted with MOLM-13-Cas9 cells transduced with luciferase-expressing lentiviral gRNAs. **e** Whole-body luminescence of mice depicted in (**f**) (*n* = 5). **f** Kaplan–Meier survival of MOLM-13-transplanted mice (*n* = 5). **g** Nuclear and cytoplasmic protein levels of SRSF1 after gRNA targeting of *SRPK1* or empty in THP1 cells. **h** Dose-response curves of AML cell lines to the SRPK1 inhibitor SPHINX31 on day 6 post-treatment (mean ± s.d., *n* = 3), reveal that cell lines driven by *MLL* rearrangements are significantly more sensitive. **i** Proliferation of THP-1 cells transduced with gRNA targeting *SRPK1* or EMPTY, and plasmids expressing a wild type (WT), a phosphomimic, a non-phosphorylatable version of SRSF1 or no cDNA (EMPTY) (mean ± s.d., *n* = 3). **j** Bioluminescence imaging of luciferase-expressing, *MLL-X*-driven, AML PDX models, treated with 2 mg/kg SPHINX31 (n = 6). Extended data in Supplementary Figure 4. **k** Kaplan–Meier survival of *MLL-X-*driven, AML PDX models, treated with 2 mg/kg SPHINX31 at indicated times (arrows) (*n* = 6). ***p < 0.001 (*t*-test). ****p < 0.0001 (*t*-test). d, day; Log-rank (Mantel–Cox) test was used for survival comparisons

Recently, we identified *SRPK1* as a cell-essential gene for AML cell lines driven by common MLL fusion genes such as *MLL-AF9* and *MLL-AF6* oncogenes^2^. Here, to validate this finding, we use lentiviral gRNAs against *SRPK1* to markedly reduce SRPK1 protein levels in Cas9-expressing AML cell lines and primary murine AMLs (Supplementary Fig. 1a–f). This was associated with differentiation (Fig. 1a, Supplementary Fig. 2a, b) and apoptosis (Fig. 1b) of AML cell lines driven by *MLL* gene fusions (*MLL-X*) or partial tandem duplication. In addition, we observed markedly reduced proliferation of these cell lines and of primary mouse AMLs driven by *MLL* fusions, whilst *MLL-WT* leukemias or non-leukemic cell lines were unaffected^13^ (Fig. 1c, Supplementary Fig. 2c). Additionally, lentiviral overexpression of gRNA-non-targetable SRPK1 cDNA, rescued the phenotype observed by disruption of *SRPK1* (Supplementary Fig. 2d, e). Also, genetic disruption of *SRPK1* by gRNA led to reduced leukemic cell growth in vivo and increased survival of immunocompromised RAIL (*Rag2*^−*/*−^*/Il2rg*^−*/*−^) mice xenotransplanted with MOLM-13-Cas9 cells (Fig. 1d–f) as well as to lower levels of nuclear SRSF1 (Fig. 1g), as previously seen with SRPK1 inhibition^4^.

To investigate the therapeutic potential of SRPK1 inhibition in AML, we used the selective SRPK1 kinase inhibitor SPHINX31^6^. We first observed that SPHINX31 inhibited the growth of *MLL*-mutant AML cell lines with an IC50  >1 order of magnitude lower than for other AML lines (Fig. 1h). This was associated with myeloid differentiation (Supplementary Fig. 2f, g), cell cycle arrest, apoptosis of human and primary mouse AMLs driven by MLL-AF9 (Supplementary Fig. 2h–k) and reduced SRSF1/2 phosphorylation and VEGF-A_165_a expression (Supplementary Fig. 3a–f), mirroring the results of genetic inhibition. Notably, ectopic expression of a phosphomimic, but not of a non-phosphorylatable SRSF1 restored THP-1 cell proliferation in association with increased nuclear SRSF1 levels, (Fig. 1i, Supplementary Fig. 3g–j) confirming the importance of SRSF1 phosphorylation in maintaining cell survival/proliferation. We also confirmed the selectivity of the SPHINX31 inhibitor for SRPK1 by performing whole kinome evaluation (Supplementary Fig. 4a, b).

To investigate the therapeutic potential of SRPK1 inhibition in AML in vivo, we determined the circulating concentration of SPHINX31 after i.p. injection. Injection of 0.8 mg/kg SPHINX31 (i.p.) into DBA2J mice resulted in a concentration of 0.225 ± 0.036 µM in plasma after 24 h. We therefore xenotransplanted RAIL mice with MOLM-13, THP-1 cells or first passage patient-derived AMLs and treated these from day 8 with 0.8 or 2.0 mg/kg of SPHINX31 or vehicle intraperitoneally, for 6 doses over 2 weeks. This led to a significant reduction in leukemic cell growth and a dose-dependent prolongation of survival of mice given MOLM-13, THP-1 and patient-derived *MLL-X* AMLs (Fig. 1j, k, Supplementary Fig. 5a–i) while the same were not observed with *MLL-WT* AMLs (Supplementary Fig. 6a–f). These data demonstrate that SRPK1 is a therapeutic vulnerability in *MLL*-rearranged AMLs.

### SRPK1 loss has no lasting effects on normal hematopoiesis

**Fig. 2:**
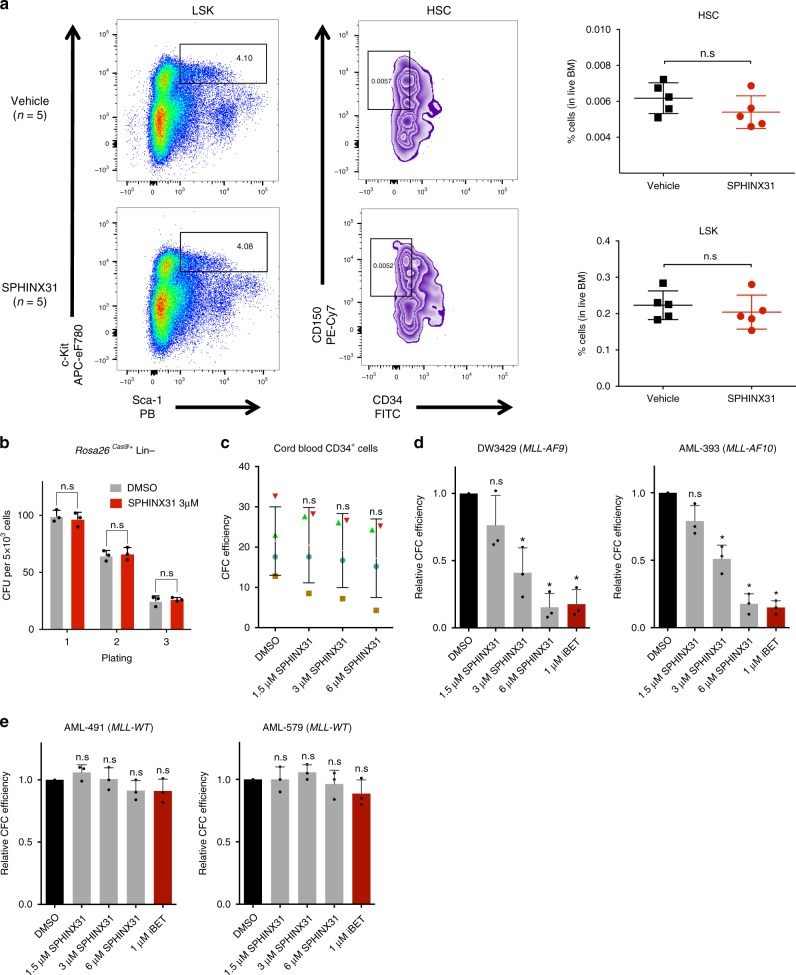
SRPK1 inhibition has no lasting effects on normal hematopoiesis. **a** Quantitation of LSK (Lin^−^/Sca1^+^/Kit^+^) and HSC (LSK/CD150^+^/CD34^−^) compartments in bone marrow from WT mice three weeks after treatment with vehicle or SPHINX31 (2 mg/kg). **b** Colony-forming assay of WT lineage negative (Lin−) HSPCs during (plating 1) and after (platings 2 & 3) treatment with 3 μM SPHINX31 (mean ± s.d., *n* = 3). **c** Colony-forming efficiency of CD34+ human cord blood cells (*n* = 4) in the presence of 1.5, 3, or 6 uM SPHINX31 (mean ± s.d., *n* = 4). These changes are not significant at the 95% confidence level according to one-way Anova on repeated measures. Error bars refer to variation across 4 different individuals (blue circle, brown square, red triangle, and green triangle). **d** Colony-forming efficiency of primary human *MLL-X* AML cells treated with 1.5, 3, or 6 μM SPHINX31 or 1 μM iBET-151 (mean ± s.d., *n* = 3 technical replicates). **e** Colony-forming efficiency of primary human *MLL-WT* AML cells treated with 1.5, 3, or 6 μM SPHINX31 or 1 μM iBET-151 (mean ± s.d., *n* = 3 technical replicates). HSC, hematopoietic stem cells; CFU, colony forming units; n.s., not significant; *p < 0.001 (*t*-test)

The drug had no lasting effects on normal hematopoiesis, as wild-type CB57BL/6N mice treated with 6 doses of SPHINX31 at 2 mg/kg over 2 weeks showed no lasting changes in the numbers of bone marrow-derived hematopoietic stem cells (HSCs), early progenitors (Lin−, Sca1+, Kit+), myeloid cells (Gr1+/Mac1+) or B-cells (B220+) and no effect on peripheral blood counts (Fig. 2a, Supplementary Fig. 7a, b). Additionally, there was no impact of SPHINX31 on the clonogenic potential of normal mouse hematopoietic stem-progenitor cells (HSPCs) despite reduced SRSF1/2 phosphorylation (Fig. 2b, Supplementary Fig. 7c). Also, we found that 1.5, 3, and 6 μM SPHINX31 did not affect the colony-forming ability of normal human cord blood CD34+ cells (Fig. 2c). However, there was strong dose-dependent inhibition of colony formation of primary human (Fig. 2d) and murine (Supplementary Fig. 7d) AML samples driven by *MLL* fusions, while there was no effect on *MLL-WT* AMLs (Fig. 2e, Supplementary Fig. 7e). Furthermore, genetic inhibition of SRPK1 using CRISPR had negligible effects on the clonogenic potential of normal mouse HSPCs despite significant reduction in SRPK1 protein levels (Supplementary Fig. 7f, g), but strongly suppressed primary murine *MLL-AF9*-driven, but not *Npm1c-*driven, AML cells (Supplementary Fig. 7h, i). These findings indicate that SRPK1 kinase activity is required for the survival of *MLL-X* AMLs, but has no lasting impact on normal hematopoiesis or the *MLL-WT* AMLs tested here.

### Inhibition of SRPK1 leads to splicing modulation

**Fig. 3:**
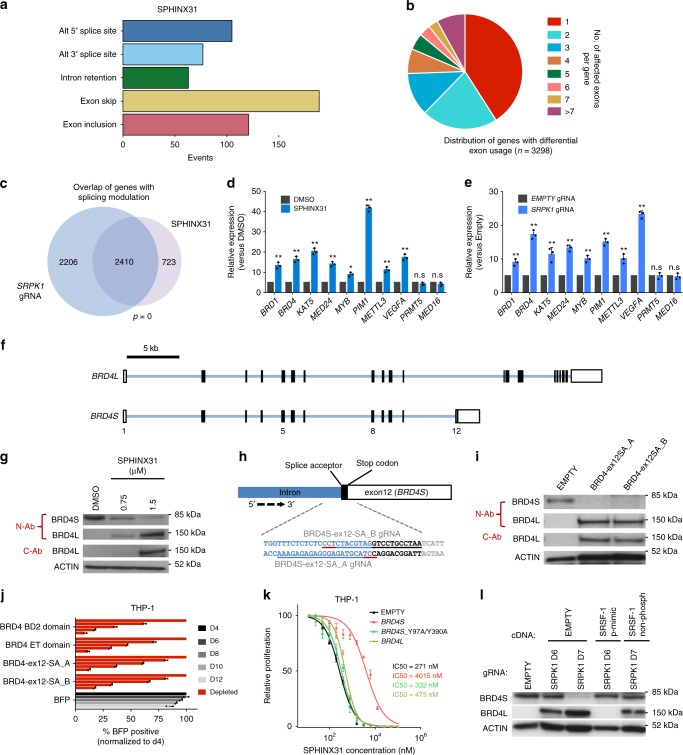
The effects of SRPK1 inhibition on global RNA splicing and *BRD4* isoform levels. **a** Frequency and type of significantly altered splicing events (FDR ≤0.001) in THP-1 cells after 24 h of treatment with 3 μM SPHINX31. **b** Number and distribution of genes with one or more differential exon usage events (FDR <0.001) in THP-1 cells after 24 h of treatment with 3 μM SPHINX31. **c** Overlap of genes with splicing changes after genetic or pharmacological inhibition of SRPK1 in THP-1 cells (hypergeometric test). **d**, **e** Quantification, by isoform-specific qRT-PCR of selected isoform changes identified upon pharmacological vs genetic inhibition of SRPK1 (mean ± s.d., *n* = 3). **f** Intron-exon structure of BRD4 long and short isoforms **g** Western blot of THP-1 cell lysates after SPHINX31 treatment (72 h), showing a marked switch from the BRD4S to the BRD4L protein isoform using both an N-terminal antibody that detects both isoforms (N-Ab) and a C-terminal Ab that detects only BRD4L (C-Ab). **h** Schematic illustration of the target sites/sequences for two gRNAs designed to specifically disrupt the *BRD4* exon 12 splice acceptor site, which defines *BRD4S* (sgRNA sequences underlined, with PAM sequence underlined in red). **i** Western blot for BRD4 in THP-1 cells transduced with each of these two gRNA display the same BRD4 isoform switch as seen with SPHINX31. **j** Competitive co-culture of THP-1 cells transfected with lentiviral gRNAs against *BRD4* (BFP positive) vs non-transfected cells normalized to %BFP on day 4 (mean ± s.d., *n* = 3). gRNAs were designed against known essential *BRD4* domains (BD2 or ET) or the splice acceptor of *BRD4S* exon 12. **k** Dose-response curves of THP-1 cells to SPHINX31 after overexpression of wild-type *BRD4L*, *BRD4S* and bromodomain mutant (Y97A/Y390A*) BRD4S* (mean ± s.d., *n* = 3). **l** Western blot for BRD4 (N-terminal antibody, N-Ab) in THP-1 cells transduced with a gRNA targeting *SRPK1* and plasmids expressing a phosphomimic version of SRSF1 cDNA or an empty control, showing block of the *BRD4S*-to-*BRD4L* isoform switch upon expression of the former. **P < 0.001 (*t*-test)

To investigate the primary effects of SRPK1 kinase inhibition on gene expression and RNA splicing we performed deep RNA-seq of THP1 cells using either SRPK1 gRNA or treatment with 3 µM SPHINX31 for 24 h. At 24 h, there was no loss of cell viability or changes in the cell cycle, but there was noticeable reduction of SRSF1/2 phosphorylation (Supplementary Fig. 8a–c). Gene set enrichment analysis of differentially expressed genes revealed enrichment for oncogenic-signatures associated with HOX gene programs (Supplementary Fig. 8d, Supplementary Data 1, 2). Splicing analysis of both pharmacological (Fig. 3a) and genetic (Supplementary Fig. 8e) inhibition of SRPK1 revealed diverse changes with exon skipping as the most common event, whilst many of the affected genes displayed significant differential usage of multiple exons (Fig. 3b, Supplementary Data 3). We compared the two splicing datasets and observed a highly significant overlap not only in the genes with altered splicing but also in specific splicing events (Fig. 3c, Supplementary Fig. 8f, Supplementary Data 4). Genes whose splicing was altered were enriched in sets associated with myeloid leukemia, chromatin modification and *MLL-*rearranged AML (Supplementary Data 5). Using isoform-specific qPCR, we confirmed significant splicing changes for several genes that were altered in both datasets including many we previously identified as cell-essential for AML^2,14^ (Fig. 3d, e). Additionally, using RNA immunoprecipitation (RIP), we found reduced SRSF1 binding to some of these transcripts after gRNA disruption and after treatment with SPHINX31 (Supplementary Fig. 8g, h). Consistent with inhibition of *MLL-X* programs, we observed *HOXA9*, *MYC* and *BCL2* downregulation after 72 h of SPHINX31 treatment (Supplementary Fig. 8i). We then focused on one of the significantly mis-spliced genes, *BRD4*, an established therapeutic target in AML^10,15^ whose two main splice isoforms have distinct epigenetic properties^12,16^. We found that SRPK1 disruption/inhibition led to a significant switch from the short (*BRD4S)* to the long (*BRD4L)* mRNA isoform, without affecting total *BRD4* mRNA (Fig. 3d–f, Supplementary Fig. 8i, j). The *BRD4S*-to-*BRD4L* switch was also seen at the protein level with both SPHINX31 and *SRPK1* gRNA in all AML cells tested irrespective of MLL mutation status (Fig. 3g, Supplementary Fig. 8k–o).

To assess the impact of the BRD4 splice switch on AML cell survival, we designed two distinct lentiviral vectors expressing gRNAs against the splice acceptor site of the final exon (exon 12, ex12-SA) of *BRD4S* (Fig. 3h). Either gRNA generated the same isoform switch to that seen with SRPK1 inhibition (Fig. 3i). Interestingly, this BRD4 isoform switching significantly reduced survival/proliferation of human *MLL-AF9* AML to a similar extent as gRNAs targeting the BRD4 second bromodomain (BD2) or extra-terminal domain (ET), previously shown to be essential for the survival of AMLs driven by *MLL* rearrangements^17^ (Fig. 3j, Supplementary Fig. 9a). In addition, we observed differentiation of THP-1 and MOLM-13 cells (Supplementary Fig. 9b), downregulation of *HOXA9*, *MYC,* and *BCL2* (Supplementary Fig. 9c, d), but not total *BRD4*, mirroring the effects of SRPK1 disruption/inhibition. Interestingly, the same experiments using *MLL*-*WT* AMLs did not show similar negative effects with the BRD4 ex12-SA guides (Supplementary Fig. 9e–g), highlighting the *MLL-X* specificity of this event. Importantly, ectopic expression of wild-type BRD4S, but not double BD1/BD2-mutant (Y97A/Y390A) BRD4S or wild-type BRD4L, significantly reduced the sensitivity of *MLL-X* cells to SPHINX31 (Fig. 3k, Supplementary Fig. 9h, i). This was not observed using *MLL*-*WT* cells (HEL) or after treating *MLL-X* cells with cytarabine (Ara-C)^18^ or daunorubicin (Supplementary Fig. 9j–m). We also showed that the *BRD4S*-to-*BRD4L* switch is dependent on SRSF1 phosphorylation as overexpression of the phosphomimic SRSF1 markedly blocked that switch (Fig. 3l). These findings strongly suggest that the switch in *BRD4* isoforms has significant anti-leukemic properties in the context of SRPK1 inhibition. Also, to rule out the possibility that *BRD4* isoform switching was a consequence (rather than the cause) of myeloid differentiation, we treated THP-1 cells with two drugs known to drive AML differentiation MI-503^19^ and cytarabine (Ara-C)^18^, and observed no effect on *BRD4* isoform ratio; whilst we also failed to see an effect upon iBET-151 treatment (Supplementary Fig. 9n). Finally, we confirmed that SRPK1 inhibition also alters BRD4 splicing in breast cancer cells (Supplementary Fig. 9o), a finding that may be of therapeutic significance in metastasis prevention^12,20^.

### SRPK1 inhibition affects BRD4 recruitment to chromatin

**Fig. 4:**
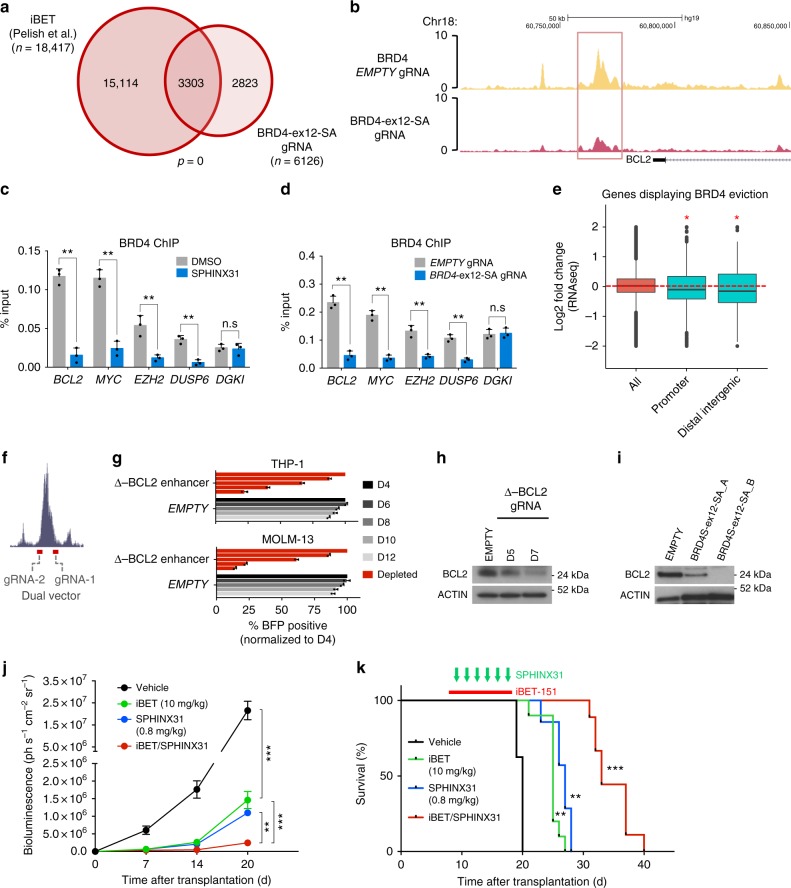
SRPK1 inhibition affects BRD4 chromatin recruitment at the BCL2 enhancer locus and synergizes with iBET-151 to inhibit growth of AML cells in vivo. **a** Overlap between loci with reduced BRD4 binding after treatment with iBET in MOLM14 from Pelish et al.^21^ and after BRD4S-to-BRD4L isoform switching by BRD4-ex12-SA_A gRNA in THP-1 cells. Both MOLM-14 and THP-1 harbor the *MLL-AF9* oncogene (Fisher’s exact test). **b** BRD4 ChIP-seq of THP-1 cells targeted with empty gRNA or gRNA BRD4-ex12-SA_A, 5 days post-transduction, showing eviction of BRD4 from the 3’ *BCL2* enhancer (left). **c**, **d** ChIP-qPCR experiments in THP-1 cells showing reduced binding of BRD4 after exposure to 1.5 μM SPHINX31 for 72 h or BRD4-ex12-SA_A gRNA (6 days post-transduction). (mean ± s.d., *n* = 3). **e** Box plot showing correlation of BRD4 eviction from promoters or linked distal intergenic regions with reduced expression of the affected genes, when compared to unselected genes genome-wide (All) (*p < 0.001; Wilcoxon test). Red, dashed line corresponds to no change in the gene expression. **f** Location of gRNAs targeting the 3’ *BCL2* enhancer. **g** Competitive co-culture showing the requirement for the *BCL2* 3’ enhancer for MOLM-13 and THP-1 cell growth and proliferation. Results were normalized to day 4 (mean ± s.d., *n* = 3). **h** Reduction of BCL2 protein in THP-1 cells by gRNA targeting of the 3’ BCL2 enhancer (**i**) and by BRD4-ex12-SA_A gRNA. **j** Quantification of luminescence for mice transplanted with luciferase-labeled THP-1 cells and treated with low dose of iBET-151 (10 mg/kg) or SPHINX31 (0.8 mg/kg) or both, showing a synergistic effect between the two drugs. **P < 0.01. ***P < 0.001. **k** Survival of mice transplanted with THP-1 cells treated as described in **j** (*n* = 8–9 animals per group). **P < 0.01. compared to vehicle (black). ***P < 0.001. compared to iBET-151(green) or SPHINX31 (blue). ***P < 0.001. Log-rank (Mantel–Cox) test was performed for the survival assays in **k**

We then questioned whether the *BRD4S*-to-*BRD4L* splicing switch has an impact on BRD4 chromatin recruitment. First, we performed BRD4 ChIP-seq using THP-1 cells transduced with *BRD4-ex12-SA*(A) gRNA or empty vector and identified 6126 differentially bound loci. Of these 6058 showed reduced BRD4 binding and the great majority of these loci were also previously reported to lose BRD4 binding upon iBET treatment^21^ (Fig. 4a, Supplementary Fig. 10a, b, Supplementary Data 6). Furthermore, 587 out of these 6058 loci were previously found to directly recruit BRD4-bound MLL fusion proteins^22,23^ (Supplementary Fig 10c). These loci include the 3’ enhancer of the *BCL2* (Fig. 4b) and *MYC* genes (Supplementary Fig 10d, Supplementary Data 7). Using BRD4 ChIP-qPCR in THP-1 and HEL cells, we confirmed BRD4 eviction from selected loci, including *BCL2*, *MYC*, and *EZH2* suggesting that BRD4S is required for BRD4 recruitment to certain chromatin sites (Fig. 4c, d, Supplementary Fig. 10e, f). In addition, we observed that genes displaying BRD4 eviction were significantly downregulated including *MYC*, *DOT1L*, *EZH2,* and *SP1* (Fig. 4e). To assess the importance of the *BCL2* 3’ enhancer in driving different AMLs we designed a dual gRNA vector targeting its flanks (Fig. 4f). CRISPR-gRNA targeting of this enhancer markedly inhibited the growth of AML cells irrespective of MLL mutation status and was associated with reduced BCL2 protein (Fig. 4g, h, Supplementary Fig. 10g–j). Notably, *BRD4* isoform switching by BRD4-ex12-SA-gRNA reduced BCL2 protein levels in *MLL-X* AML, but not in *MLL-WT* (Fig. 4i, Supplementary Fig. 10k), further highlighting that the *BRD4S*-to-*BRD4L* switch is a selective vulnerability of the former. These results indicate that *BRD4S* suppression by SRPK1 inhibition affects BRD4 recruitment to chromatin and is associated with downregulation of key MLL-X fusion mediators, including *BCL2* and *MYC*.

Finally, as SRPK1 inhibition impacts on BRD4 function as do BET inhibitors (iBETs)^10,24^, we set out to test if their anti-leukemic properties are redundant or additive, particularly as iBETs inhibit multiple BET family members beyond BRD4^24^. To evaluate this, we employed an 8 (SPHINX31) by 6 (iBET) dose matrix in THP-1 cells and we analyzed the combination effect using the Bliss independence model^25^. We observed that there is a strong synergistic effect between the two drugs across a clinically relevant range of iBET doses (Supplementary Fig. 11a). We verified this by showing that 200 nM iBET-151 markedly reduced the IC50 of SPHINX31 against THP-1 and MOLM-13 (Supplementary Fig. 11b). To examine if this combination is effective in vivo we tested the impact of the combination in the RAIL xenotransplantation model and found strong synergy, with significant deceleration of AML growth and prolongation of mouse survival (Fig. 4j, k, Supplementary Fig. 11c), without noticeable toxicity. We also performed the same experiments using the *MLL-WT* HEL cells and did not observe any synergistic effect either in vitro or in vivo (Supplementary Fig. 11d–f).

## Discussion

Taken together our findings indicate that the splicing kinase SRPK1 is a novel, druggable therapeutic vulnerability of AMLs driven by several recurrent *MLL* rearrangements^26^. Phosphorylation of SRSF1 is required for dissociation of its RNA recognition motif from its own RS domain^27^ and from CLK1^28^, enabling free SRSF1 to recruit U1 snRNP to the 5’ splice site. Failure to recruit U1 snRNP and any associated effects on splicing or spliceosome assembly can then alter splice site as well as polyadenylation choice^29^ as observed here for BRD4. Whilst SRPK1 inhibition led to changes in mRNA isoform levels of many genes, the impact on BRD4 isoforms was striking and the genetic recapitulation of this event in isolation using *BRD4-ex12-SA* gRNA significantly inhibited the growth of *MLL*-rearranged AMLs (Supplementary Fig. 11g), in association with suppression of target oncogenes including *MYC* and *BCL2*. Additionally, the anti-leukemic effects of the SRPK1 inhibitor SPHINX31 were strongly inhibited by ectopic expression of BRD4S further suggesting that the BRD4 isoform switch is one key mediator of the anti-leukemic effects of this compound. The fact that SRPK1 loss/inhibition leads to isoform switching rather than full inhibition of BRD4, may underlie its specificity to *MLL*-rearranged AMLs in contrast to the effects of direct BRD4 inhibitors which have broader anti-leukemic properties^30^, although the mechanisms for this specificity need to be fully established in future studies. Furthermore, SPHINX31 and i-BET-151 showed synergistic anti-AML effects without noticeable toxicity in mice, pointing to a possible clinical use of SRPK1 inhibitors, either alone, or in combination with bromodomain inhibitors.

Recent studies have shown that modulation of splicing is a promising therapeutic approach in leukemias with mutations in genes encoding spliceosomal proteins^31–33^. Our findings extend the applicability of this approach to AMLs lacking such mutations and also propose that inhibition of SRPK1 should be investigated in the treatment of other malignancies where BRD4 isoform balance plays a role, such as metastatic breast cancer^34^, in which SRPK1 is also an important mediator^20^.

## Methods

### Cell culture

MOLM-13, THP-1, NOMO-1, EOL-1, HEL, K562, and HL-60 were cultured in RPMI1640 (Invitrogen) supplemented with 10% FBS (PAA) and 1% penicillin/streptomycin/glutamine. OCI-AML2 and OCI-AML3 were cultured in alpha-MEM (Lonza) supplemented with 20% FBS (PAA) and 1% penicillin/streptomycin/glutamine. HPC-7 was cultured in IMDM (Invitrogen) supplemented with 10% FBS, 100 ng ml^−1^ SCF (Peprotech), 7.48 × 10^−5^ M 1-thioglycerol (Sigma), 1% penicillin/streptomycin/glutamine. 32D was cultured in RPMI1640 (Invitrogen) supplemented with 10% FBS, 10 ng ml^−1^ IL3 (Peprotech) and 1% penicillin/streptomycin/glutamine. 293T, MDA-MB-231 and BT-474 cells were cultured in DMEM (Invitrogen) supplemented with 10% FBS (PAA) and 1% penicillin/streptomycin/glutamine. All cancer cell lines were obtained from the Sanger Institute Cancer Cell Collection and negative for mycoplasma contamination. Human cell lines employed were either not listed in the cross-contaminated or misidentified cell lines database curated by the International Cell Line Authentication Committee (ICLAC) or were previously verified by karyotyping.

### Lentivirus production and transduction

Lentiviruses were produced in HEK293 cells using ViraPower Lentiviral Expression System (Invitrogen) according to manufacturer's instructions. 1 × 10^6^ cells and viral supernatant were mixed in 2 ml of culture medium supplemented with 8 μg ml^−1^ (human) or 4 μg ml^−1^ (mouse) polybrene (Millipore), followed by spinfenction (60 min, 900 *×* *g*, 32 °C) and further incubated overnight at 37 °C. The medium was refreshed on the following day and the transduced cells were cultured further.

### gRNA competition assays

gRNA competition assays were performed using single and dual gRNA vectors^2^. For the validation of individual target genes, one gRNA was derived from the CRISPR library used in the screens and another gRNA was designed using http://www.sanger.ac.uk/htgt/wge/. Viral supernatants were collected 48 h after transfection. All transfections and viral collections were performed in 24-well plates and transduction was performed as mentioned above. For gRNA/BFP competition assays, flow cytometry analysis was performed on 96-well plates using a LSRFortessa instrument (BD). Gating was performed on live cells using forward and side scatter, before measuring of BFP^+^ cells. The gRNA sequences are listed in Methods.

### Isolation of hematopoietic progenitors

*Flt3*^*ITD/+*^ mice were kindly provided by Gary Gilliland and crossed with *Rosa26*^*Cas9/+*^ mice. Freshly isolated bone marrow from 6- to 10-week-old female *Rosa26*^*Cas9/+*^, *Flt3*^*ITD/+*^*; Rosa26*^*Cas9/+*^ or moribund *Npm1*^*flox−cA/+*^*; Flt3*^*ITD/+*^, *Npm1*^*flox−cA/+*^*; Nras*^*G12D/+*^ mice were used. Bone marrow cells were exposed to erythrocyte lysis (BD PharmLyse, BD Bioscience), followed by magnetic bead selection of Lin^−^ cells using the Lineage Cell Depletion Kit (Miltenyi Biotec) according to the manufacturer’s instructions. Lin^−^ was cultured in X-VIVO 20 (Lonza) supplemented with 5% BIT serum (Stem Cell Technologies) 10 ng ml^−1^ IL3 (Peprotech), 10 ng ml^−1^ IL6 (Peprotech) and 50 ng ml^−1^ of SCF (Peprotech). Retrovirus constructs pMSCV-MLL-AF9-IRES-YFP and pMSCV-MLL-ENL-IRES-Neo were used with package plasmid psi-Eco to produce retrovirus. 293T cells (Life Technologies) were cultured and prepared for transduction in 10 cm plates as described above. For virus production, 5 μg of the above plasmids and 5 μg psi-Eco packaging vector were transfected dropwise into the 293T cells using 47.5 μl TransIT LT1 (Mirus) and 600 μl Opti-MEM (Invitrogen). The resulting viral supernatant was harvested and transduction of primary mouse cells was performed in 6-well plates as mentioned above. After transduction, transduced cells were sorted for YFP (for MLL-AF9) or selected with neomycin (for MLL-ENL).

For in vivo experiments related to Fig. 2, 6–10-week-old female *Rosa26*^*Cas9/+*^ mice were treated triweekly for two weeks with either vehicle or 2 mg/kg SPHINX31 (Exonate). Four weeks post-treatment, bone marrow cells from these mice were freshly extracted (as mentioned above) and blocked with anti-mouse CD16/32 (BD Pharmigen, cat. no. 553142) and 10% mouse serum (Sigma). For the identification of LK/LSK, LT-HSC, myeloid and B-cell subpopulations, staining was performed using CD4 PE/Cy5 (Biolegend, cat. no. 100514), CD5 PE/Cy5 (Biolegend, cat. no. 100610), CD8a PE/Cy5 (Biolegend, cat. no. 100710), CD11b PE/Cy5 (Biolegend, cat. no. 101210), B220 PE/Cy5 (Biolegend, cat. no. 103210), TER-119 PE/Cy5 (Biolegend, cat. no. 116210), GR-1 PE/Cy5 (Biolegend, cat. no. 108410), SCA-1 Pacific Blue (Biolegend, cat. no. 122520), CD150 PE/Cy7 (Biolegend, cat. no. 115913), CD34 FITC (BD Pharmigen, cat. no. 553733) and CD117 APC-eFluor780 (eBioscience, cat. no. 47-1171). In each of the multi-colour flow cytometry experiments, we included the fluorescence minus one (FMO) controls. FMO controls provide a measure of spillover in a given channel. This allows for correct gating and selects only the stained cells in the experimental sample. Flow cytometry analysis was performed using a LSRFortessa instrument (BD) and resulting data were subsequently analyzed using FlowJo.

For blood counts, 20 μl of blood was collected from the tail-vein of the mice using a capillary pipette containing anticoagulants (EDTA). The EDTA anti-coagulated blood samples were used to obtain a complete blood count using a VetABC analyzer (Horiba ABX). Samples were counted no longer than five minutes after blood was drawn.

For replating assays using the SRPK1 inhibitor, 5000 lineage negative cells and primary murine AML cells were plated in three wells of 6-well-plate of M3434 methylcellulose (Stem Cell Technologies) in the presence of 3 µM SPHINX31. For replating assays using SRPK1 gRNA, 5000 lineage negative cells and primary murine AML cells were plated in three wells of 6-well-plate of M3434 methylcellulose (Stem Cell Technologies) after selection with 1.0 μg ml^−1^ puromycin for 3 days starting from day 2 post-transduction. The colonies were counted 7 days later and further 5000 cells re-seeded and re-counted after a week until the 3rd replating.

### Flow cytometry analyses of AML cells

Cells were transduced with gRNA vectors or treated with SPHINX31 and stained at the indicated time points with anti-mouse CD11b PE/Cy5 (Biolegend, cat. no. 101210) and anti-human CD11b PE (eBiosciences, cat. no. 9012-0118) or anti-human CD13 FITC (eBioscience, cat. no. 11-0138-42). Data were analyzed by using LSRFortessa (BD) and FlowJo.

Apoptosis levels were measured in human and/or mouse AML cells transduced with dual gRNA vectors (against *SRPK1* and 3’ *BCL2* enhancer) and/or treated with 1 or 3 μM SPHINX31 (Exonate) at indicated time points, by using Annexin V (Life Technologies, cat. no. V13242). Data were analyzed by using LSRFortessa (BD) instruments.

Cell cycle stages were measured in human and/or mouse AML cells transduced with dual gRNA vectors against *SRPK1* and/or treated with 1 or 3 μM SPHINX31 (Exonate) at indicated time points, using Propidium Iodide from Abcam (ab14083). Data were analyzed using LSRFortessa (BD) instruments.

### Drug and proliferation assays

All suspension cells were plated (96-well) in triplicate at 5000–10,000 cells per well and treated for 72 h with vehicle or the indicated concentrations of SPHINX31 (0.04-50 μM, Exonate), Cytarabine (0.075–40 μM, Sigma), Daunorubicin (0.075–80 μM, Selleckchem) and indicated IC20 doses of iBET-151 (0.04–25 μM, Selleckchem). On day 3, plates were measured (for treatments with Cytarabine and Daunorubicin) using CellTiter 96 AQueous Non-Radioactive Cell Proliferation Assay (Promega) in order to calculate the relative cell proliferation. Regarding the treatment with SPHINX31, an equal volume for all wells was split-back with fresh media and compound, such that the resulting cell density in each well matched the initial seeding density. Plates were measured on day 6 using CellTiter 96 AQueous Non-Radioactive Cell Proliferation Assay (Promega) in order to calculate the relative cell proliferation. All the compounds were dissolved in DMSO.

For synergy studies between SPHINX31 and iBET, THP-1 cells were seeded in 96-well plates at 10,000 cells per well and treated with SPHINX31 (dose range of 0.039–5 μM) and iBET (dose range of 9.8–312.5 nM) in an 8 by 6 matrix. Each treatment was carried out in triplicate. Cells were treated for 72 h, and cell viability was determined using CellTiter 96 AQueous Non-Radioactive Cell Proliferation Assay (Promega) in order to calculate the relative cell proliferation. Cell viability for each treatment was normalized against the DMSO control group. A Bliss independence model was employed to evaluate combination effects and calculate the Bliss independence score^3^. All the compounds were dissolved in DMSO.

### Rescue of phenotype assays

THP-1 cells were transduced with the lentiviral cDNA constructs pKLV2-*SRPK1*-PURO, pKLV2-TY1-*SRSF1*-PURO, pKLV2-*SRSF1*(ΔN/RS)-PURO (non-phosphorylatable SRSF1, truncated before the SRSF1 SR repeat domains), an empty pKLV2 vector control or with a phosphomimic SRSF1 cDNA construct^27^. The *SRPK1* cDNA of the pKLV2-*SRPK1*-PURO vector was modified at the PAM sequence (Chr 6: 35,921,124 - Human GRCh38) with a change from G to C, retaining the Arginine residue (CGC instead of CGG) in order to prevent the cutting through the SRPK1 gRNA-3.

Transduced cells were selected for puro and further transduced with lentiviral SRPK1 or empty gRNA. Competition assays of gRNAs/BFP-positive cells were performed using flow cytometry analysis on 96-well plates using a LSRFortessa instrument (BD). Gating was performed on live cells using forward and side scatter, before measuring of BFP^+^ cells. The gRNA sequences are listed in Table 1.

**Table 1:** List of primer sequences

gRNA competition assay	
**Human gRNAs**	**Sequence**
*SRPK1* (1)	GGTGTGGATGATACGGCACT
*SRPK1* (2)	CTGCATGGTATTTGAAGTTT
*SRPK1* (3)	TTACCGGTCTCACCATGGAG
BRD4S-ex12-SA_A	TTTCTCTCTCCCTCTACGT
BRD4S-ex12-SA_B	TTAGGCAGGACCTACGTAG
BRD4_ET_domain	AGTCGATTTCAATCTCGTCG
BRD4_BD2_domain	GTAGAAGGGCCAGGCGTAGG
BCL2 Intergenic (1)	GAGTGTCTCAATGGGCAGCG
BCL2 Intergenic (2)	AAGAGCCACGGCCTAAAGCA
**Mouse gRNAs**	**Sequence**
*Srpk1* (1)	ACCTGCAGACCCCGATGGTG
*Srpk1* (2)	TGAATGAGCAGTACATTCGA

	RT-PCR	
	Forward Primer	Reverse Primer
*HOXA9*	GAATGAGAGCGGCGGAGAC	GAGCGAGCATGTAGCCAGTTG
*c-MYC*	AATGAAAAGGCCCCCAAGGTAGTTATCC	GTCGTTTCCGCAACAAGTCCTCTTC
*BCL2*	CTGCACCTGACGCCCTTCACC	CACATGACCCCACCGAACTCAAAGA
*BRD4*	CTCCTCCTAAAAAGACGAAGA	TTCGGAGTCTTCGCTGTCAGAGGAG
*GAPDH*	GATGCCCTGGAGGAAGTGCT	AGCAGGCACAACACCACGTT

	RIP qPCR	
	Forward Primer	Reverse Primer
*BRD1*	ACATCCCATGGACTTTGCCACA	CTCACCGCGGCTCTATAGAACA
*BRD4*	CTCCTCCTAAAAAGACGAAGA	TTCGGAGTCTTCGCTGTCAGAGGAG
*MYB*	AGCACCGATGGCAGAAAGTACT	TCTCCCCTTTAAGTGCTTGGCA
*MED24*	AGGAGCTCAAGTGGACAGCTTT	GGTGAGCTTCAGCAGGAACTCA

	ChIP qPCR	
	Forward Primer	Reverse Primer
*BCL2*	ACAGCGCCAACAGAACTACT	CCCCACAACGGAGCTGTAAT
*MYC*	CAAGCTCTCCACTTGCCCCT	GCCCTGAGATGTGTCTGCCT
*EZH2*	CTTCTGAGTCCCACCGGGTG	GCCGTGTGTTCAGCGAAAGA
*DUSP6*	GTAGAGGAAGGTCGGGGAGA	CACACAGGGCCATCTCAACT
*DGKI*	GCCACCCCCTCATCTCTCAC	TCTTCCAAGGACCCAGGGGA

Splicing Validation – RT-qPCR		
Exon Skipping	Forward Primer	Reverse Primer
*BRD1 (Exons 11-12)*	AATGTCACTGAGGTCGCTGG	ACGTGCTGAAGATTGGGGAG
*BRD4-Long (Exons 10-12)*	TCGGAGCCATCTCTGTTTC	CGACTTTGAGACCCTGAAGC
*BRD4-Short (Exons 10-12)*	AATGATTAGGCAGGACCTGTT	CGACTTTGAGACCCTGAAGC
**Exon Inclusion**	**Forward Primer**	**Reverse Primer**
*MYB (Exon 8-Intron 8)*	TGAGCTAAAAGGACAGCAGGT	CAAAGCACAAGGAGCCATC
**Alternative 5' Splice Site**	**Forward Primer**	**Reverse Primer**
*PIM1 (Exon 4-Intron 4)*	CGACATCAAGGACGAAAACA	AGACACCCACACCCTTTCCT
**Intron Retention**	**Forward Primer**	**Reverse Primer**
*MED24 (Exon 6-Intron 6)*	AAGTCCCTGGGATGTGTGC	CTGGAGAAAACCCTCAGCAG
*KAT5 (Exon 1- Intron 1)*	CGGAACCAGGACAACGAAGA	ACCTCTCGGAGCAGCTAAGA
*METTL3 (Exon 9-Intron 8)*	AGACCCTGGTTGAAGCCTTG	TGGGGCCCAATTCAATAGGT
*VEGFA (Exon 5-Intron 5)*	ACCAAAGAAAGATAGAGCAAGACAA	ATTGTTGCTGCCACCACAAG
*MED16 (Exon 14-Intron 14)*	GACTTGAGCATGGTGACACAG	AAAGTGGGGCAGGCGTAA
*PRMT5 (Exon 12-Intron 11)*	CACCCATTCCCTCATGTCTG	TTTCCTTAACATCTCTCCTTACCTT

Furthermore, transduced cells were selected for BFP, further treated with SPHINX31 at the indicated doses, in 96-well plates. An equal volume for all wells was split-back with fresh media and compound, such that the resulting cell density in each well matched the initial seeding density. Plates were measured on day 6 using CellTiter 96 AQueous Non-Radioactive Cell Proliferation Assay (Promega) in order to calculate the relative cell proliferation.

THP-1 or HEL cells were electroporated in Buffer R (Invitrogen) with plasmids encoding the WT *BRD4S* isoform (1–722 aa), the full-length WT *BRD4* (*BRD4L)*, the *BRD4S* isoform mutated for both bromodomains (Y97A/Y390A) or an empty vector as a control^35^. In each replicate 150,000–250,000 cells were electroporated with 500 ng of each plasmid. Electroporation was performed using the Neon Transfection System (Thermo Fisher Scientific). Electroporation conditions used for THP-1 and HEL cells were based on manufacturer instructions (1350V, 35 ms, 1pulse). 2 days after electroporation, THP-1 and HEL cells were plated (96-well) in triplicate at 10,000 cells per well and treated for 72 h with vehicle or the indicated concentrations of SPHINX31 (0.04–50 μM, Exonate), Cytarabine (0.075–40 μM, Sigma) or Daunorubicin (0.075–80 μM, Selleckchem). On day 3, an equal volume for all wells was split-back with fresh media and compound, such that the resulting cell density in each well matched the initial seeding density. Plates were measured on day 6 using CellTiter 96 AQueous Non-Radioactive Cell Proliferation Assay (Promega) in order to calculate the relative cell proliferation. All the compounds were dissolved in DMSO.

### Adult primary leukemia and cord blood sample drug and proliferation assays

All human AML and cord blood samples were obtained with informed consent under local ethical approval (REC 07-MRE05-44). Primary human AML cells or cord-blood-derived CD34^+^ cells were tested for colony-forming efficiency in StemMACS HSC-CFU semi-solid medium (Miltenyi Biotec) in the presence of the indicated concentration of SPHINX31 or DMSO. Colonies were counted by microscopy 11–12 days (AML cells) or 12–14 days (CD34^+^ cells) after plating.

### Western blot analysis

Cells were treated with indicated concentrations of SPHINX31 or transduced with dual/single lentiviral gRNA or an empty vector and selected with 1.0 μg ml^−1^ puromycin for 3 days starting from day 2 post-transduction. The transduced cells were further cultured for 2 days before lysis. Cell pellets were resuspended in whole cell lysis buffer (50 mM Tris-HCl, pH = 8, 450 mM NaCl, 0.1% NP-40, 1 mM EDTA), supplemented with 1 mM DTT, protease inhibitors (Sigma), and phosphatase inhibitors (Sigma). Protein concentrations were assessed by Bradford assay (Bio-Rad) and an equal amount of protein was loaded per track. Prior to loading, the samples were supplemented with SDS-PAGE sample buffer and DTT was added to each sample. 10–40 μg of protein was separated on SDS-PAGE gels, and blotted onto polyvinylidene difluoride membranes (Millipore). All the uncropped, full-size scans of western blots are presented in Supplementary material.

### Chromatin immunoprecipitation and quantitative PCR (ChIP-qPCR) analysis

THP-1 and HEL cells were treated for 24 h with either DMSO (0.1%, vehicle) or SPHINX31 (3 μM). Firstly, THP-1 and HEL cells were cross-linked with 1% formaldehyde for 10 min. 20 × 10^6^ THP-1 or HEL cells were used for each immunoprecipitation with 3 ug of specific antibodies or IgG, 6 days post-transduction with gRNAs or 24 h after SPHINX31 treatment. Cells were resuspended in ChIP Lysis Buffer (1%SDS, 10 mM EDTA, 50 mM Tris-HCl, pH = 8, protease inhibitors) and sonicated in Bioruptor Pico (Diagenode) for 10 cycles. Sonicated chromatin was diluted 1:10 in modified RIPA buffer (1% Triton; 0.1% deoxycholate; 90 mM NaCl; 10 mM Tris-HCl, pH 8; EDTA free protease inhibitors) and incubated overnight with 3 μg of anti-BRD4 (C-term) from Bethyl Laboratories (A301-568A) and 3 μg IgG Isotype Control from Abcam (ab171870). Next protein A/G (50% A 50% G) Dynabeads (Invitrogen) were added to the chromatin and incubated 2 h at 4 °C followed by magnetic separation. Beads were subsequently washed twice with mixed micelle buffer (150 mM NaCl, 0.2% SDS, 20 mM Tris-Cl, pH 8.0, 5 mM EDTA, 5.2% sucrose, 1% Triton X-100); high salt buffer (250 mM NaCl, 5 mM Tris-Cl, pH 8.0, 0.5 mM EDTA, 0.05% sodium deoxycholate, 25 mM HEPES pH 8.0, 0.5% Triton X-100) and LiCl buffer (250 mM LiCl, 10 mM Tris-Cl, pH 8.0, 10 mM EDTA, 0.5% NP40-Nonidet, 0.5% sodium deoxycholate) and once with elution buffer (1%SDS; 100 mM NaHCO_3_). Beads were then resuspended in elution buffer supplemented with DNAse free RNAse (Roche, #11119915001). Cross-linking was reverted by the incubation at 37 °C for 30 min followed by the incubation at 65 °C overnight. Immunoprecipitated DNA was purified with ChIP DNA Clean & Concentrator Columns (Zymo) and analyzed on an ABI 7900 real-time PCR machine, Fast SybrGreen PCR mastermix according to the manufacturer’s instructions. Primer sequences are listed in Table 1. Experiments were performed as paired biological triplicates, with single cultures split for treatment in each replicate experiment.

### RNA immunoprecipitation and quantitative PCR (RIP-qPCR) analysis

THP-1 cells harboring the lentiviral cDNA construct pKLV2-TY1-SRSF1-PURO were homogenized in adequate volumes of polysome lysis buffer (10 mM HEPES-KOH (pH 7.0), 100 mM KCl, 5 mM MgCl_2_, 25 mM EDTA, 0.5% IGEPAL, 2 mM dithiothreitol (DTT), 0.2 mg/mL Heparin, 50 U/mL RNase OUT (Life Technologies), 50 U/mL Superase IN (Ambion) and 1 × complete protease inhibitor tablet (Roche). The suspension was centrifuged at 14,000 × *g* at 4 °C for 10 min to remove debris. Lysates containing 1 mg protein were incubated with 500 ng normal IgG (Cell Signalling Technologies, #2729) or anti-TY1 (Diagenode, C15200054), at 4 °C overnight on an inverse rotator. Protein A–sepharose beads (Life Technologies, 50 μL per tube) were first blocked in NT2 buffer (50 mM Tris-HCl (pH 7.5), 150 mM NaCl, 1 mM MgCl_2_ and 0.05% IGEPAL) supplemented with 5% BSA, 0.02% sodium azide and 0.02 mg/mL heparin at 4 °C for 1 h, and then added into the lysates followed by a 3-h incubation at 4 °C on an inverse rotator. The beads were subsequently washed five times in NT2 buffer. RNAs were released by incubating in proteinase K buffer (50 mM Tris (pH 8.0), 100 mM NaCl, 10 mM EDTA, 1% SDS and 1 U/mL proteinase K) for 30 min at 65 °C, and pelleting by adding an equal volume of isopropanol and centrifuging at 12,000 g at 4 °C for 10 min. After washing once with 75% ethanol, RNAs were reverse-transcribed into first-strand cDNA and used for real-time RT–PCR analysis to detect the indicated mRNAs. Data were normalized to IgG control groups.

### Nuclear/cytoplasmic protein fractionation

THP-1 cells were either transduced with a phosphomimic *SRSF1* cDNA or a dual lentiviral *SRPK1* gRNA as well as an empty vector and selected with 1.0 μg ml^−1^ puromycin for 3 days starting from day 2 post-transduction. The transduced cells were lysed on day 5 post-transduction and protein fractionation (nuclear/cytoplasmic) was performed using the PARIS fractionation kit from Ambion (AM1921).

### Antibodies

Western blot experiments were performed using the following antibodies: anti-SRPK1 from Abcam (ab90527, with 1:1000 working dilution), anti-SRSF1 from Abcam (ab38017, with 1:2000 working dilution), anti-pSRSF from EMD-Millipore (MABE50, with 1:1000, working dilution), anti-ACTIN from Abcam (ab8227, with 1:5000 working dilution), anti-BRD4 (N-term) from Abcam (ab128874, with 1:1000 working dilution), anti-BRD4 (C-term) from Bethyl Laboratories (A301-985A, with 1:1000 working dilution), anti-MYC from Abcam (ab32, with 1:1000 working dilution), anti-BCL2 from Abcam (ab32124, with 1:1000 working dilution) and anti-TBP from Abcam (ab51841, with 1:2000 working dilution). For the ChIP experiments, the following antibodies were used: anti-BRD4 (C-term) from Bethyl Laboratories (A301-568A) and IgG Isotype Control from Abcam (ab171870).

### Quantitative RT-PCR

Total RNA was isolated from AML cells using the RNeasy Mini or Micro Kit (Qiagen). For cDNA synthesis, total RNA was reverse-transcribed with the SuperScript VILO cDNA Synthesis kit (Life Technologies). The levels of specific RNAs were measured using the ABI 7900 real-time PCR machine and the Fast SybrGreen PCR mastermix according to the manufacturer’s instructions. *BRD4*, *HOXA9*, *MYC*, and *BCL2* mRNA levels were normalized to, the housekeeping gene, *GAPDH*.

Validation of the splicing modulation after SRPK1 inhibition (gRNA or SPHINX31) was measured by quantification of the isoform changes compared to the non-edited/non-treated cells. *BRD4* isoform switching was defined by the relative enrichment of the long isoform levels compared to the short isoform levels in either treated vs untreated or transduced vs non-transduced conditions.

To determine the effect of IC50 SPHINX31 on *BRD4* isoform switching, in comparison to IC50 of other anti-leukemic drugs iBET-151 (Selleckchem), MI-503 (Active Biochem) and Cytarabine (Selleckchem), quantification of the relative enrichment of the long isoform compared to the short isoform of *BRD4* was performed, after treatment of the cells for 72 h.

All samples, including the template controls, were assayed in triplicate. The relative number of target transcripts was normalized to GAPDH expression in the same sample. The relative quantification of target gene expression was performed with the standard curve or comparative cycle threshold (*C*_T_) method. The primer sequences are listed at the end of the Methods section.

### May–Grunwald–Giemsa and cytospin staining

10^5^ cells were cytospun for 5 min at 300 × *g* onto glass slides. Slides were then stained for 3 min with May–Grunwald solution (Sigma-Aldrich) at room temperature. After washing in water, they were incubated for 20 min in Giemsa solution (Sigma-Aldrich) (1:20 in water). Slides were washed again in water before being mounted with Mowiol embedding medium.

### Histological analyses

Mice were euthanized, autopsied and the dissected spleen tissue samples were fixed in 4% paraformaldehyde, dehydrated, and embedded in paraffin. Paraffin blocks were sectioned at 4 μm and stained with hematoxylin and eosin (H&E). Images were acquired using a MIRAX Slide Scanner (Zeiss).

### Generation of PDX models

Six- to ten-week-old NSG female mice were injected with 10^6^ patient-derived AML cells by intravenous injection. Indicated doses of SPHINX31 or vehicle were delivered to the mice via intraperitoneal injection (IP) on day 10 post-transplant, triweekly for two weeks (6 treatments). Indicated doses of vehicle or SPHINX31 were delivered to the mice via intraperitoneal injection (IP) on day 10 post-transplant. SPHINX31 was dissolved in 20%(w/v) 2-hydroxypropyl-beta-cyclodextrin vehicle (Sigma, H107). At day 10 post-transplant, tumor burdens of animals were detected using IVIS Lumina II (Caliper) with Living Image version 4.3.1 software (PerkinElmer). Briefly, 100 μl of 30 mg/ml D-luciferin (BioVision) was injected into each animal intraperitoneally. Ten min after injection, the animals were maintained under general anesthesia by isoflurane and put into the IVIS chamber for imaging. The detected tumor burdens were measured and quantified by the same software. Diseased mice were identified by qualified animal technicians from the Sanger mouse facility. All animal studies were carried out in accordance with the Animals (Scientific Procedures) Act 1986, Amendment Regulations (2012) UK under project license PBF095404. Randomization and blinding were not applied.

### Whole-body bioluminescent imaging

For in vivo experiments, MOLM-13, THP-1 and HEL cells expressing Cas9 were first transduced with a firefly luciferase-expressing plasmid (System Biosciences). After propagation, the cells were transduced with a dual lentiviral gRNA vector expressing either empty or SRPK1 gRNA (day 0) and selected with puromycin from day 2 to day 5. At day 5 post-transduction, the cells were suspended in fresh medium without puromycin. At day 6, 1 × 10^5^ cells were transplanted into a Rag2^−/−^ Il2rg^−/−^ mouse by tail-vein injection. For the in vivo drug experiments related to Fig. 1 and Supplementary Figure 4, MOLM-13 and THP-1 cells were transduced with a firefly luciferase-expressing plasmid (System Biosciences). 1 × 10^5^ cells were transplanted into a Rag2^−/−^ Il2rg^−/−^ mouse by tail-vein injection. Indicated doses of SPHINX31 or vehicle were delivered to the mice via intraperitoneal injection (IP) on day 10 post-transplant, triweekly for total two weeks (6 treatments). For the in vivo drug experiments related to Fig. 4 and Supplementary Figure 5, THP-1 and HEL cells were transduced with a firefly luciferase– expressing plasmid (System Biosciences). 1 × 10^5^ cells were transplanted into a Rag2^−/−^ Il2rg^−/−^ mouse by tail-vein injection. Indicated doses of vehicle, SPHINX31 and/or iBET-151 were delivered to the mice via intraperitoneal injection (IP) from day 10 post-transplantation. Both SPHINX31 and iBET-151 were dissolved in 20%(w/v) 2-hydroxyproply beta-cyclodextrin vehicle (Sigma, H107).

At day 10 post-transplant, the tumor burdens of the animals were detected using IVIS Lumina II (Caliper) with Living Image version 4.3.1 software (PerkinElmer). Briefly, 100 μl of 30 mg/ml D-luciferin (BioVision) was injected into the animals intraperitoneally. Ten min after injection, the animals were maintained in general anesthesia by isoflurane and put into the IVIS chamber for imaging. The detected tumor burdens were measured and quantified by the same software. Diseased mice were assessed blindly by qualified animal technicians from the Sanger mouse facility. All animal studies were carried out in accordance with the Animals (Scientific Procedures) Act 1986, Amendment Regulations (2012) UK under project license PBF095404. Randomization and blinding were not applied.

### SPHINX31 pharmacokinetics

Three Dba/2J mice were given i.p. injections of 0.8 mg/kg SPHINX31 and sacrificed after 24 h when blood was taken by cardiac puncture into EDTA tubes. Plasma was isolated by centrifugation, and an equal volume (100 µl) acetonitrile added. An internal standard of 100 µg/ml of a related compound (compound 3 from Batson et al) was added to samples to account for any loss of material during preparation. The solutions were centrifuged for 15 min at 4 °C and the supernatant taken for analysis. Solutions were evaporated at 37 °C for eight hours and resuspended in 30 µl acetonitrile ready for analysis by LC MS, using a Waters 2795 HPLC system. Detection was achieved by positive ion electrospray (ESI + ) mass spectrometry using a Waters Micromass ZQ spectrometer in single ion monitoring (SIM) mode, at 352 *m/z* units ([M+H]^+^). Chromatography (flow rate 1 mL·min^−1^) was achieved using a Phenomenex Kinetex column (2.6 μ, C_18_, 100 Å, 4.6 × 50 mm) equipped with a Phenomenex Security Guard precolumn (Luna C_5_ 300 Å). Peaks occurring at these times in the SIM chromatograms per compound were integrated using Water MassLynx software. The chromatograms produced clear peaks at the expected molecular weights. The integrated area under the peaks and read from a standard curve led to quantification of the circulating concentration of SPHINX31.

### Kinome analysis

Kinase binding assay for 489 kinases was carried out by KINOMEscan, DiscoverX, at 1 µM SPHINX31. To identify potential inhibition of kinases other than SRPK1, a truncated version of SRPK1 was used in the screen, which does not contain part of the loop that SPHINX31 binds to, so the SRPK1 activity will not show positivity in this assay. The percent inhibition of kinase-substrate interaction is determined and the red spots correspond to the kinases where there is more 50% inhibition.

Radioactive kinase assays were carried out by the MRC Dundee Kinase Centre for SRPK1, SRPK2, CLK1 and CLK2 from 10 µM to 0.0003 µM SPHINX31 with ATP at the Km for each kinase.

### SPHINX31 SRSF1 phosphorylation

1 × 10^6^ cells/ml, unless otherwise stated, were treated with SPHINX31 at 1% DMSO for 48 h then lysed in buffer containing 50 mM Hepes, 150 mM NaCl, 0.5% Triton-X100, 1 mM EDTA, 1 mM PMSF, 10 mM Na_3_VO_4_, 10 mM NaF and protease inhibitor cocktail (Roche). 50 µg protein was separated on SDS-PAGE gels and immunoblotted with anti-SRSF1 from Abcam (ab38017), anti-pSRSF from EMD-Millipore (MABE50) and anti-ACTIN from Abcam (ab8227).

### VEGF enzyme-linked immunosorbent assay (ELISA)

VEGF_165_a capture antibody, at a concentration of 0.25  μg/ml, was incubated on high-binding 96-well plates overnight at room temperature. The plates were blocked (1% BSA in PBS) and serial dilutions of recombinant human (rh)VEGF165 standards (ranging from 500 pg/ml to 1.95 pg/ml) were added, incubated alongside sample lysates, typically 200 μg in 100 μl per well. The plate was incubated for 2 h at room temperature with shaking, washed and incubated with 100 μl/well of biotinylated goat anti-human VEGF (0.1 μg/ml; R&D systems) for 2 further hours at room temperature. After washing, 100 μl/well of Horseradish Peroxidase (HRP)-conjugated streptavidin (1:200; R&D Systems) was added and plates were left at room temperature for 20 min. The plates were washed and color change induced with substrate A and B (DY-999; R&D Systems) for 1 h under light protection. The reaction was stopped by addition of 100 μl/well of 1 M HCl and the absorbance was read immediately in an ELISA plate reader (Dynex Technologies Opsys MR system plate reader) at 450 nm with a control reading at 620 nm. A standard curve was calculated from mean absorbance values of standards enabling the estimation of VEGF concentration for each sample.

### RNA-seq analysis

For the experiment of the pharmacological inhibition, THP-1 cells were treated for 24 h with DMSO or 3 uM SPHINX31, followed by RNA extraction. For the experiment of the genetic inhibition, THP-1 cells were transduced with a dual lentiviral gRNA vector expressing either empty or SRPK1 gRNA (day 0) and selected with puromycin from day 2 to day 5. At day 5 post-transduction, the cells were suspended in fresh medium without puromycin. At day 6 cells were harvested for RNA extraction. RNAseq data from both experiments were generated as biological triplicates and the 75 bp paired-end Illumina reads were aligned using STAR to the human genome (hg19). Furthermore, reads that were aligned to multiple locations in the genome, or marked as duplicates by Picard, or not aligned as a proper read pair according to SAMtools were removed from further analysis.

The total number of reads that align to the exons of each gene in the human genome as defined by GENCODE version 19 were obtained using HTSeq. Using DESeq2 we obtained expression fold changes (FC) and False Discovery Rates (FDRs) for genes between the above-mentioned two conditions. The genes that were differentially expressed between these two conditions (padj ≤0.01) are given in Supplementary Data 1.

The enrichments of C2 (curated gene-sets) and C6 (oncogenic-signatures) from MSigDB in our gene-sets were computed using hypergeometric testing. Functional enrichment profiles for the downregulated genes (log_2_FC ≤ -1) are given in Supplementary Data 2. The test statistic values for the genes obtained from DESeq2 were used to rank the genes and Gene Set Enrichment Analysis (GSEA) was performed for the C6 positional datasets.

Differential exon and splice junction usages between the two conditions were computed using JunctionSeq, which uses the popular DEXSeq to compute statistical significance. Splice junctions with FDR ≤ 0.001 were annotated as Alternative 5’ splice sites, Alternative 3’ splice sites, Intron retention, Exon inclusion, Exon skipping and Alternative Transcript End sites. The frequencies of occurrences of these Alternative Splicing Events (ASEs) are given in Fig. 3a and Supplementary Figure 8e. If one or both of the splice sites in a given splice junction overlaps with splice sites of transcripts annotated as “retained intron” or “non-sense mediated decay” by Ensembl, the splice junction is annotated as “intron retention”. A splice junction (SJ_1_) is termed as exon skipping or inclusion if there exists an in-between exon. If the mean fold change of the splice junctions that share the splice sites with SJ_1_ is greater than 1 and the fold change of SJ_1_ is less than 1, then SJ_1_ is annotated as an “Exon skipping” (ES) event and if the vice-versa occurs then SJ_1_ is annotated as an “Exon inclusion” (EI) event.

Genes that show differential splicing (FDR ≤ 0.0005), Supplementary Data 3) are further tested for functional enrichment of C2 and C6 gene sets using hypergeometric testing and the results are tabulated in Supplementary Data 5.

### ChIP-seq data analysis

The ChIP-seq reads for BRD4 binding upon *BRD4S*-to-*BRD4L* isoform change using gRNA (BRD4g) or upon transduction with an empty gRNA, were aligned to hg19 using BWA aligner. Duplicate reads were removed using Picard from further analysis and peaks were called using MACS2 using default parameters. The ChIP-seq was performed in duplicates for each condition and the aligned reads along the peaks were then used to obtain differential binding sites using DiffBind. A site was termed as differential if the FDR ≤ 0.1 and the fold changes along with FDR is given in Supplementary Data 6. The 6058 differential peaks were mapped to the genes using ChIPSeeker and distribution of peaks across the genome is given in Supplementary Fig 10a, b. In order to obtain the tag density profile for the 6058 regions that were differentially downregulated upon *BRD4S*-to-*BRD4L* isoform change, replicates were merged and normalized tag densities were generated (bigWig) including the tag densities for *BCL2* and *MYC* loci shown in Fig. 4b and Supplementary Fig 10d.

The ChIP-seq data performed by Pelish et al.^21^ in duplicates for BRD4 binding in MOLM-14 cell line upon DMSO or iBET treatment (BRD4-iBET) and downloaded from Gene Expression Omnibus (GSM1893934 to GSM1893941). The sequenced reads were aligned, processed and peaks called using the above-mentioned method. Only the overlapping peaks between the replicates were retained for identification of differential binding sites of BRD4 binding upon iBET treatment (500 nM, 6 h). A peak is termed as differential if the fold change is better than 1.5 and the FDR ≤ 0.1 and these peaks were mapped to the genes using ChIPSeeker.

The high-confidence binding sites of MLL-AF9 fusion and MLL-WT proteins in THP-1 cell line were obtained from Prange *e*t al^23^. The overlaps of these peaks with the 6058 differential peaks upon BRD4 isoform change (see above) are presented in Supplementary Fig 10c.

### Statistical analysis

Statistical analyses performed were specified in figure legends. Differences were considered significant for *P*-values < 0.05. The significances of the overlaps of the gene sets were computed using hypergeometric tests using R and the significances of the overlaps of peak sets were calculated using Fisher’s exact test using BedTools.

## Electronic supplementary material

Supplementary Information

Description of Additional Supplementary Files

Supplementary Data 1

Supplementary Data 2

Supplementary Data 3

Supplementary Data 4

Supplementary Data 5

Supplementary Data 6

Supplementary Data 7

## Data Availability

The datasets used in this study have been deposited to the European Nucleotide Archive and can be accessed from ENA: ERP104309. The other data that support the findings of this study are available within the article or its supplementary information.
