# Supplementary material for: SRPK1 maintains acute myeloid leukemia through effects on isoform usage of epigenetic regulators including BRD4
Source: Nat Commun. 2018 Dec 19;9:5378. doi: 10.1038/s41467-018-07620-0 (PMC6300607; doi:10.1038/s41467-018-07620-0)
Supplement: Supplementary file 2 — Description of Additional Supplementary Files [file 41467_2018_7620_MOESM2_ESM.pdf]

Description of Additional Supplementary Files

File Name: Supplementary Data 1
Description: Differential gene expression analysis of THP-1 cells after 24 hours of treatment with 3µM SPHINX31 compared to vehicle treated control

File Name: Supplementary Data 2
Description: Gene Set Enrichment Analysis of significantly downregulated genes after 24 hours treatment of THP-1 cells with 3µM SPHINX31

File Name: Supplementary Data 3
Description: Splice site changes identified in THP-1 cells after transduction with SRPK1 gRNA compared to empty control.

File Name: Supplementary Data 4
Description: Overlap of significant splicing events between genetic and pharmacological inhibition of SRPK1

File Name: Supplementary Data 5
Description: Gene Set Enrichment Analysis of genes with significant splicing modulation after 24 hours treatment of THP-1 cells with 3µM SPHINX31

File Name: Supplementary Data 6
Description: Total and differential peaks identified in THP-1 cells after BRD4S-to BRD4L isoform switching using gRNA compared to empty control

File Name: Supplementary Data 7
Description: Overlapping loci illustrating MLL-AF9 recruitment and BRD4 eviction after BRD4S-to BRD4L isoform switching
