# Supplementary material for: SRPK1 maintains acute myeloid leukemia through effects on isoform usage of epigenetic regulators including BRD4
Source: Nat Commun. 2018 Dec 19;9:5378. doi: 10.1038/s41467-018-07620-0 (PMC6300607; doi:10.1038/s41467-018-07620-0)
Supplement: Supplementary file 1 — Supplementary Information [file 41467_2018_7620_MOESM1_ESM.pdf]

Supplementary Information

SRPK1 maintains acute myeloid leukemia through effects on isoform usage of epigenetic regulators including BRD4

Tzelepis et al.

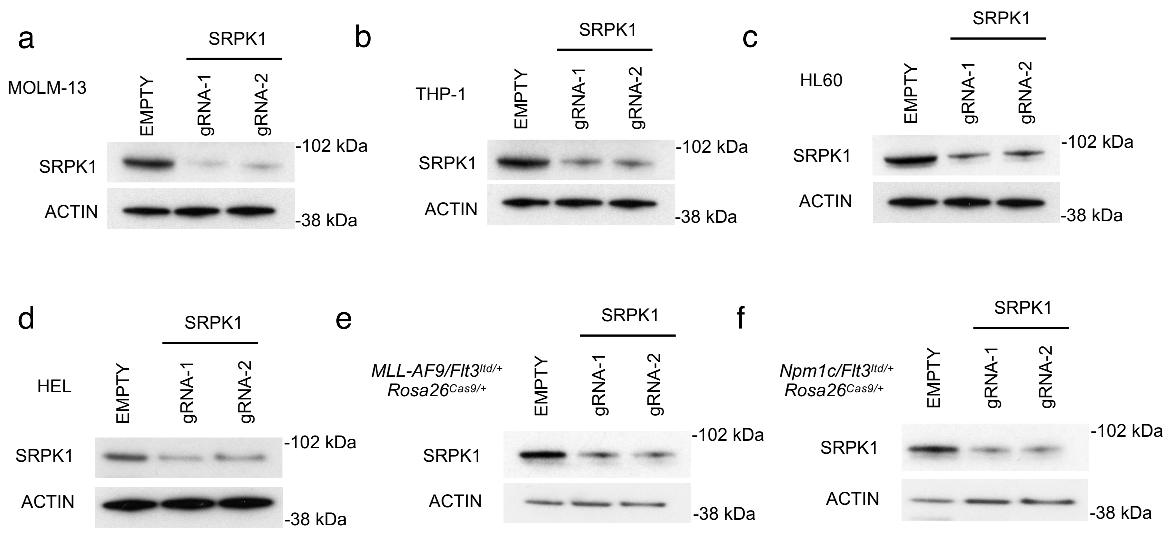

Supplementary Figure 1: CRISPR targeting of SRPK1 leads to efficient loss of total SRPK1 protein levels in human and primary mouse AML

(a-d) Western blots in human AML cell lines, showing a marked reduction of SRPK1 protein levels after CRISPR targeting of *SRPK1* with two independent gRNAs. **(e-f)** Western blots in primary mouse AML cells, showing a marked reduction of SRPK1 protein levels after CRISPR targeting of *Srpk1* with two independent gRNAs.

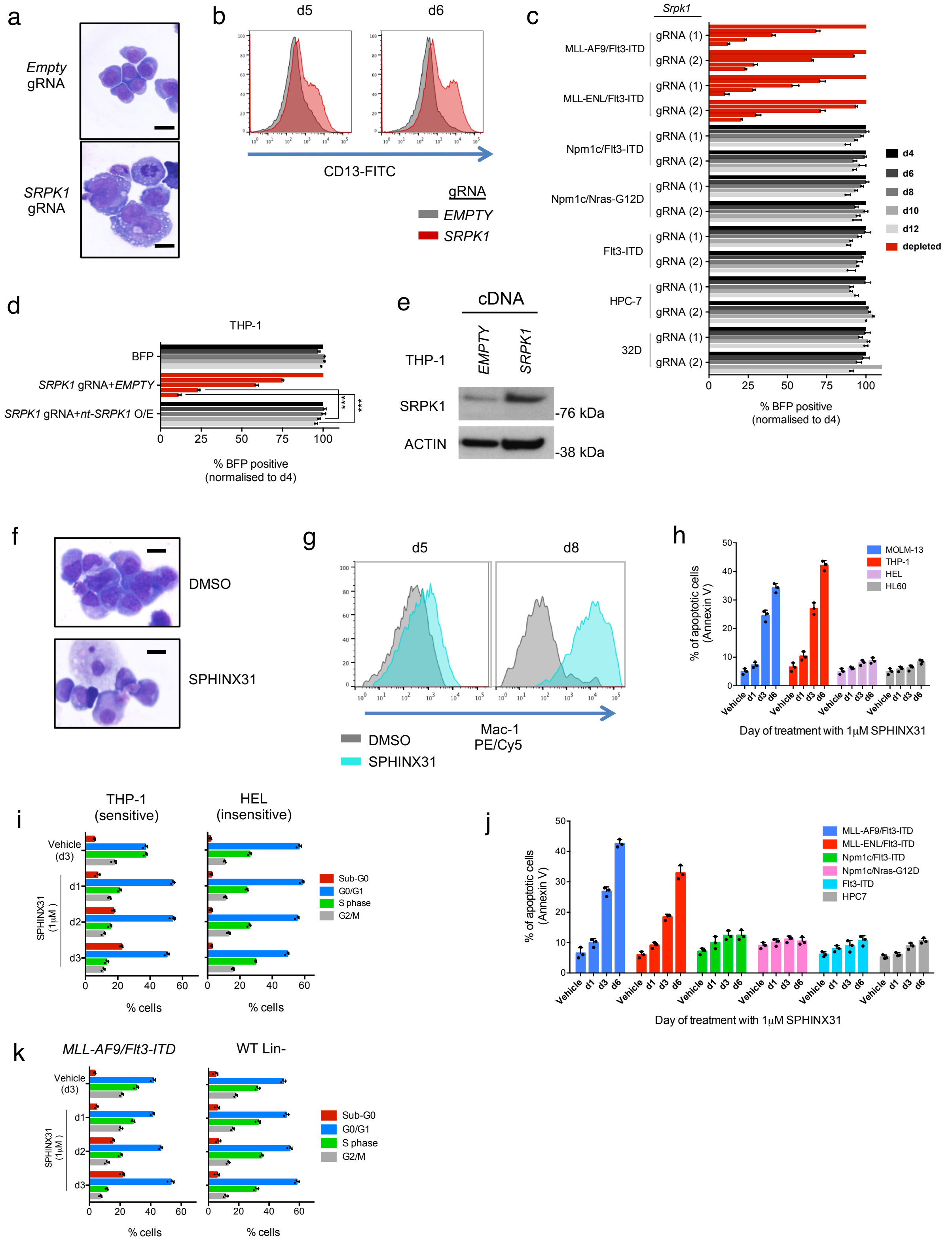

Supplementary Figure 2: Extended analysis of the impact of SRPK1 inhibition on human and mouse AMLs

(a) Morphology of MOLM-13 cells on day 6 after transduction with a gRNA for SRPK1 or an empty vector. **(b)** *CD13* expression in “empty” or *SRPK1* gRNA targeted MOLM-13 cells. **(c)** Competitive co-culture of transduced vs non-transduced murine hematopoietic cells after SRPK1 gRNA delivery by lentivirus. The non-leukemic hematopoietic stem cell lines HPC7

and 32D were used as control. Results are normalized to day 4 (mean ± s.d., n=3). **(d)** Competitive co-culture assay of THP-1 cells transduced with a gRNA targeting *SRPK1* and plasmids expressing a gRNA-non-targetable version of *SRPK1* cDNA (nt-SRPK1, see methods) or an empty control, showing rescue of the negative phenotype upon overexpression of SRPK1. Results are normalized to day 4 (mean ± s.d., n=3). **(e)** Western blot in THP-1 cells, showing a marked increase of SRPK1 protein levels upon transduction with an SRPK1 cDNA construct. **(f)** Morphology and **(g)** Mac-1 expression of primary murine *MLL-AF9/Flt3*$^{ITD/+}$ cells after treatment with 3µM SPHINX31 for 72 hours. **(h)** Apoptosis in human AML cells 1, 3, and 6 days after 72 hours of treatment with 1µM SPHINX31 compared to vehicle (day 3) (mean ± s.d., n=3). **(i)** Cell cycle analysis of THP-1 and HEL cells (negative control) treated with 1µM SPHINX31 (mean ± s.d., n=3). SPHINX31 reduced the number of cells in S and increased those in the G0/G1 phases for the THP1 but not the HEL line. **(j)** Apoptosis in murine cells 1, 3, and 6 days after 72 hours of treatment with 1µM SPHINX31 compared to vehicle (day 3) (mean ± s.d., n=3). **(k)** Cell cycle analysis of primary murine *MLL-AF9/Flt3*$^{ITD/+}$ and WT lineage negative (Lin-) HSPCs after treatment with 1µM SPHINX31 for 72 hours (mean ± s.d., n=3). SPHINX31 reduced the number of cells in S and increased those in the G0/G1 phases for the primary murine *MLL-AF9/Flt3*$^{ITD/+}$ cells but not the WT lineage negative (Lin-) HSPCs. ***, p<0.001 (t-test); d, day, scale bars in **(a)** and **(e)** correspond to a length of 10 µm. *** P<0.001 (t-test).

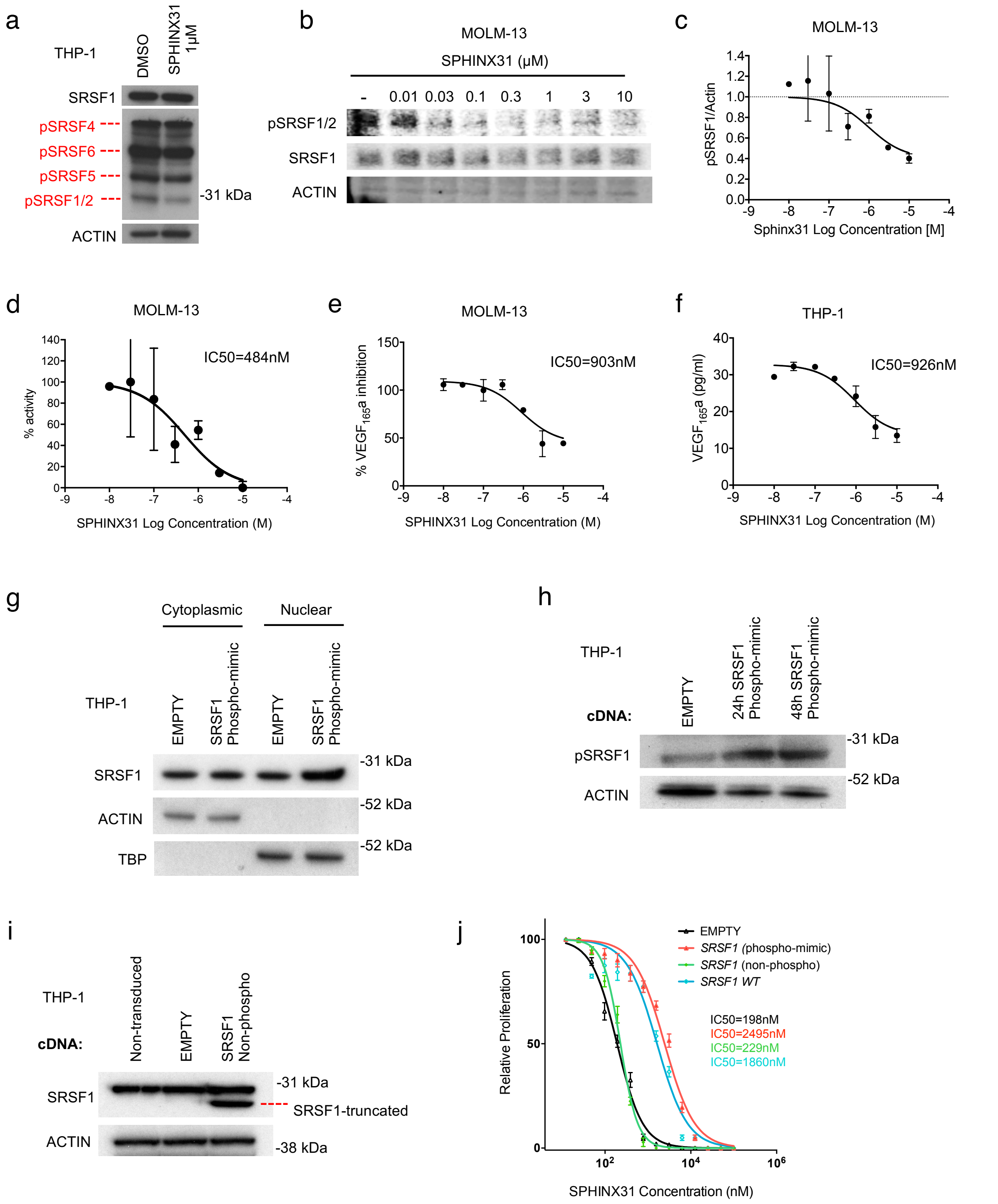

Supplementary Figure 3: Treatment of human AML cells with SPHINX31 leads to loss of SRSF1 phosphorylation and production of VEGF-A$_{165}$a

(a) Western blots for total SRSF1 and panPhospho-SRSF (pSRSF) in THP-1 cell lysates after treatment with 1μM SPHINX31 for 48 hours. Bands corresponding to phosphorylated SRSF1/2, SRSF4, SRSF5 and SRSF6 are indicated. SRSF1 and SRSF2 proteins have the same size. **(b)** Immunoblot of SRSF1-immunoprecipitate from MOLM-13 cells treated with increasing concentrations of SPHINX31. Blots were probed with antibodies to phospho-SR peptide, SRSF1 or ACTIN respectively. **(c)** Quantification of 3 immunoblots showing a concentration dependent reduction in SRSF1 phosphorylation with SPHINX31. **(d)**

Normalization of the pSRSF1 levels (vs total SRSF1) to calculate the IC50 of SPHINX31. **(e,f)** ELISA for VEGFA-165 levels using isoform-specific antibodies shows a SPHINX31 concentration dependent reduction in VEGFA165 in MOLM-13 and THP-1 cells. **(g)** Nuclear and cytoplasmic protein levels of SRSF1 in THP1 cells transduced with plasmids expressing a phospho-mimic SRSF1 version or an empty control, showing increase of the nuclear levels of SRSF1 upon overexpressing of the phospho-mimic SRSF1. **(h)** Western blots for phospho-SRSF1 (pSRSF ab) and ACTIN in THP-1 cells transduced with plasmids expressing a phospho-mimic or an empty control, showing increase of the SRSF1 phosphorylation upon overexpressing of the phospho-mimic SRSF1. **(i)** SRSF1 protein levels in THP1 cells transduced with plasmids expressing a non-phosphorylatable SRSF1 version (truncated) or an empty control, showing increase of the SRSF1 upon overexpressing of the non-phosphorylatable SRSF1. **(j)** Dose-response curves of THP-1 cells to SPHINX31 after transduction with plasmids expressing a full-length (WT), a phospho-mimic or a non-phosphorylatable version of SRSF1 (mean ± s.d., n=3).

a

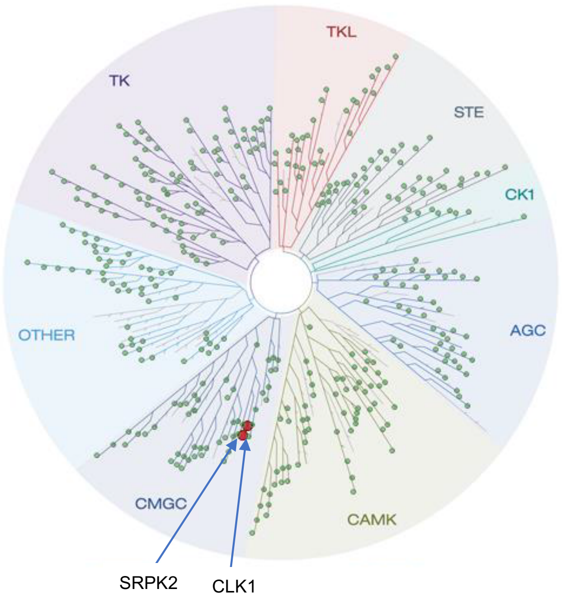

b

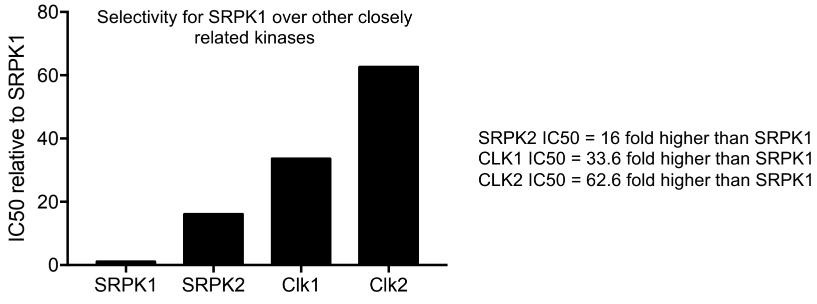

Supplementary Figure 4: Whole kinome evaluation of the SPHINX31 activity

(a) Kinase binding assay for 489 kinases was carried out by KINOMEscan, DiscoverX, at 1 μM SPHINX31. To identify potential inhibition of kinases other than SRPK1, a truncated version of SRPK1 was used in the screen, which does not contain part of the loop that SPHINX31 binds to, so the SRPK1 activity will not show up in this assay. Red circles indicate

>50% inhibition of kinase binding. **(b)** Selectivity of SPHINX31 for SRPK1 over closely related kinases. Selectivity was determined by radioative ATP bidning assay (Dundee Screening service).

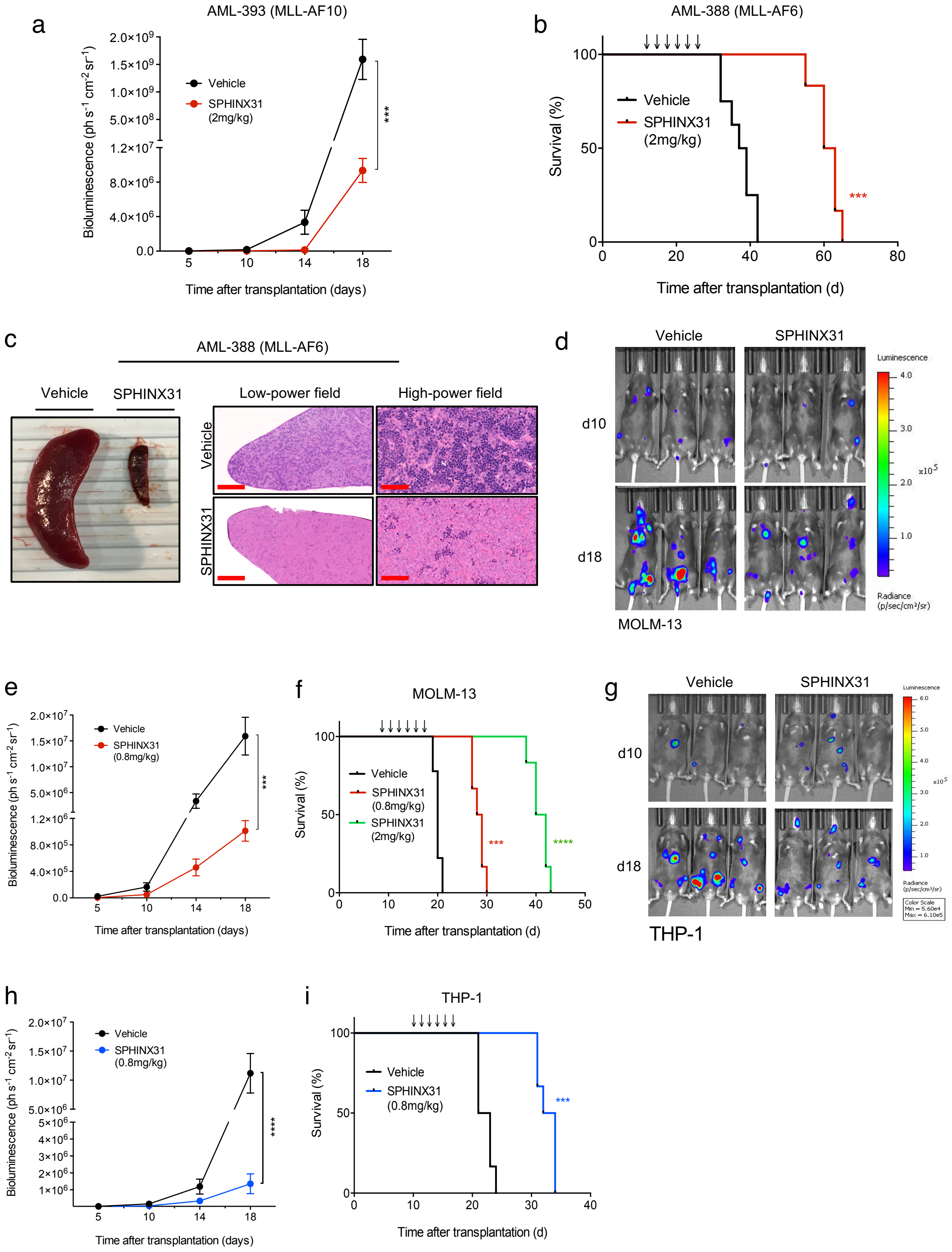

Supplementary Figure 5: SPHINX31 treatment of *MLL-X* AMLs impairs leukemia expansion *in vivo*

(a) Quantification of luminescence volume from Figure 1j. **(b)** Kaplan-Meier survival of *MLL-X*-driven, AML PDX models treated with 2mg/kg SPHINX31 at indicated times (arrows) (n=6). **(c)** Differences in splenic size and H&E staining of sections from *MLL-X* PDX models shown in b. Scale bars, 1 mm (low-power field) and 100 µm (high-power field). **(d)** Bioluminescence

imaging of mice transplanted with luciferase-labeled MOLM-13 cells and treated with SPHINX31 (0.8mg/kg) (n=6) **(e)** Quantification of luminescence from **(d)**. **(f)** Kaplan-Meier survival of mice transplanted with MOLM-13 cells and treated with six doses (arrows) of 0.8 or 2mg/kg SPHINX31 or vehicle (n=6 per group). **(g)** Bioluminescence imaging of mice transplanted with luciferase-labeled THP-1 cells and treated with six doses (arrows) of SPHINX31 (0.8mg/kg) or vehicle (n=6 per group). **(h)** Quantification of luminescence from **(g)**. **(i)** Kaplan-Meier survival of mice transplanted with THP-1 cells and treated with six doses of 0.8 mg/kg SPHINX31 at indicated times (arrows) (n=6). ***, $p<0.001$ (t-test). ****, $p<0.0001$ (t-test). d, day; Log-rank (Mantel-Cox) test was performed for survival studies.

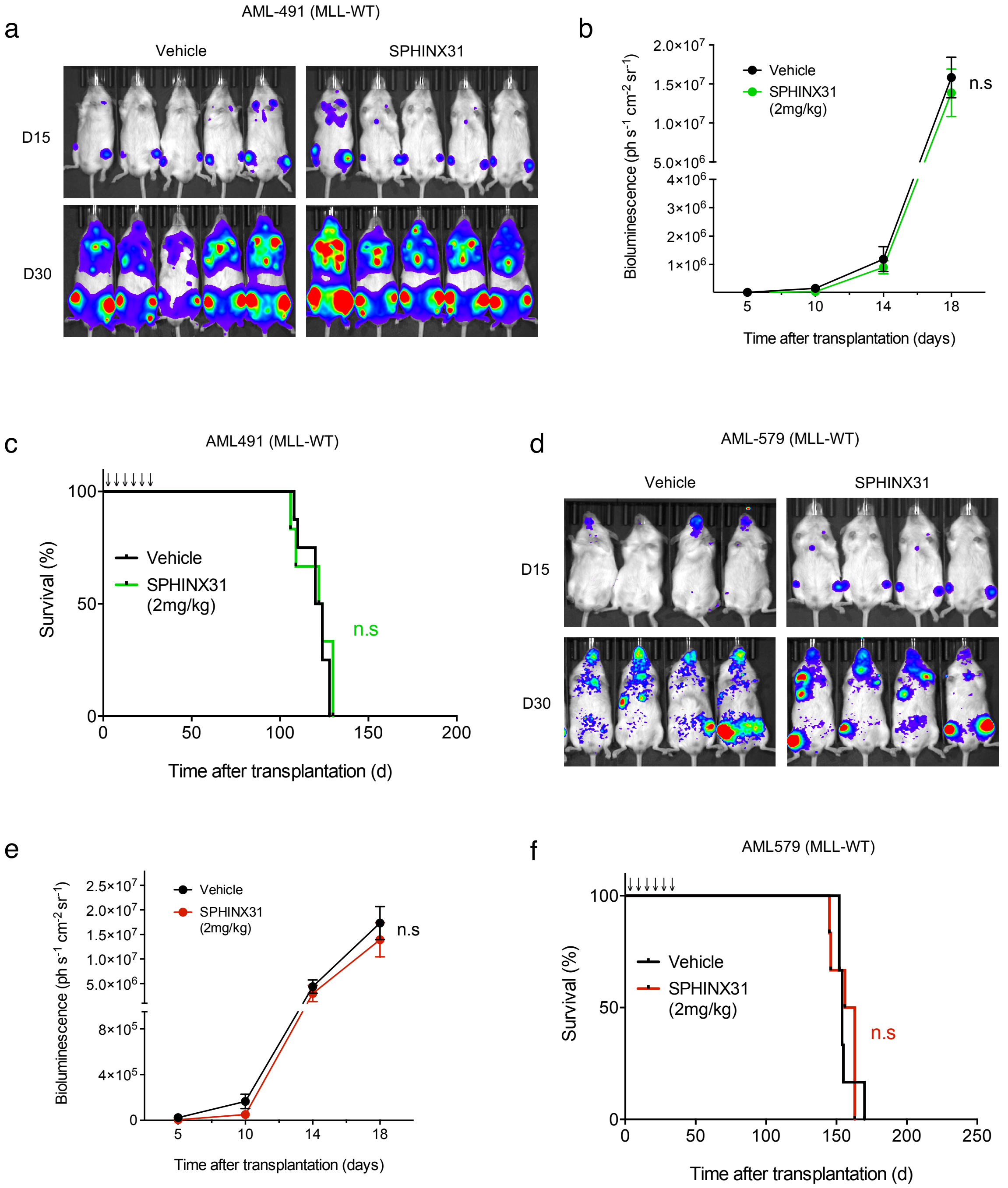

Supplementary Figure 6: SPHINX31 treatment of *MLL-WT* AMLs has no discernible effect on leukemia expansion *in vivo*

(a) Bioluminescence imaging of *MLL-WT*, AML PDX models (AML-491) treated with 2mg/kg SPHINX31 (n=6). **(b)** Quantification of luminescence from **(a)**. **(c)** Kaplan-Meier survival of *MLL-WT*, AML PDX models (AML-491) treated with six doses of 2mg/kg SPHINX31 at indicated times (arrows) (n=6). **(d)** Bioluminescence imaging of an additional *MLL-WT*, AML PDX models (AML-579) treated with 2mg/kg SPHINX31 (n=6). **(e)** Quantification of luminescence from **(d)**. **(f)** Kaplan-Meier survival of *MLL-WT*, AML PDX models (AML-579) treated with six doses of 2mg/kg SPHINX31 at indicated times (arrows) (n=6). ***, $p<0.001$ (t-test). ****, $p<0.0001$ (t-test). d, day; Log-rank (Mantel-Cox) test was performed for survival

studies.

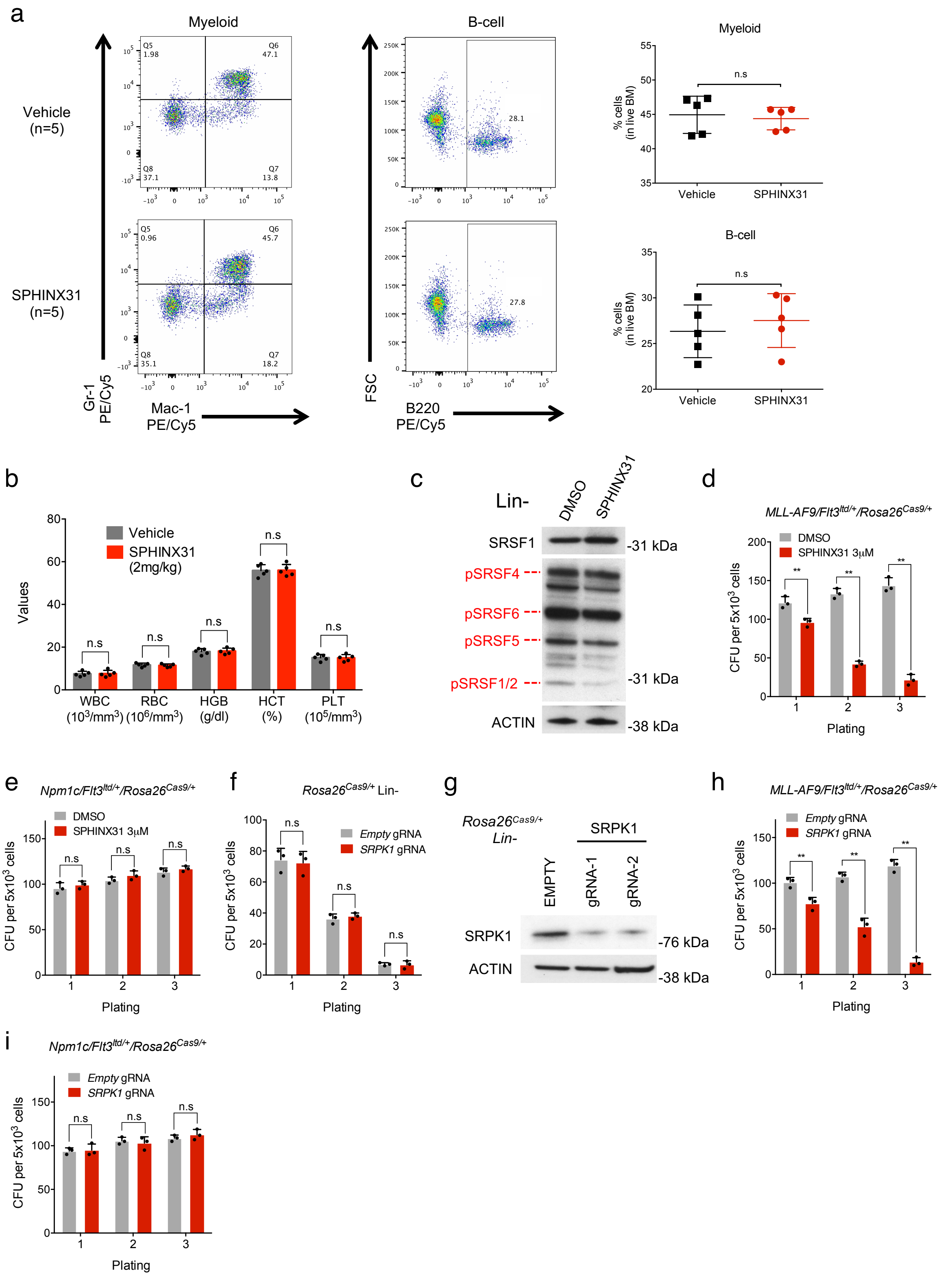

Supplementary Figure 7: SPHINX31 does not affect the numbers of myeloid cell and B lymphocytes in WT mice

(a) Quantitation of myeloid (Gr1/Mac1) and B-cell (B220) compartments identified in bone

marrow from WT mice three weeks after treatment with vehicle or SPHINX31 (2mg/kg) (n=5). **(b)** Blood count results for the mice from **(a)**, treated with either vehicle or SPHINX31 (2mg/kg). **(c)** Western blots for total SRSF1 and panPhospho-SRSF (pSRSF) in primary murine Lin- cells after treatment with 3μM SPHINX31 for 48 hours. Bands corresponding to phosphorylated SRSF1, SRSF2, SRSF4, SRSF5 and SRSF6 are indicated. SRSF1 and SRSF2 proteins have the same size. **(d)** Colony forming efficiency of primary murine *MLL-AF9/Flt3*$^{ITD/+}$*/Rosa*$^{Cas9/+}$ AML cells treated with 3μM of SPHINX31 (mean ± s.d., n=3 technical replicates). **(e)** Colony forming efficiency of primary murine *NPM1c/Flt3*$^{ITD/+}$*/Rosa*$^{Cas9/+}$ cells treated with 3μM of SPHINX31 (mean ± s.d., n=3 technical replicates). **(f)** Colony forming assay of Lin- cells from Rosa26$^{Cas9/+}$ transduced with "Srpk1" vs "empty" gRNA (mean ± s.d., n=3). **(g)** Western blot in Lin- cells from Rosa26$^{Cas9/+}$, showing a marked reduction of SRPK1 protein levels after CRISPR targeting of *Srpk1* with two independent gRNAs. **(h)** Colony forming assay of primary murine *MLL-AF9/Flt3*$^{ITD/+}$*/Rosa*$^{Cas9/+}$ cells transduced with "Srpk1" vs "empty" gRNA (mean ± s.d., n=3). **(i)** Colony forming assay of primary murine *NPM1c/Flt3*$^{ITD/+}$*/Rosa*$^{Cas9/+}$ cells transduced with "Srpk1" vs "empty" gRNA (mean ± s.d., n=3). n.s., not significant; **, $p<0.001$ (t-test).

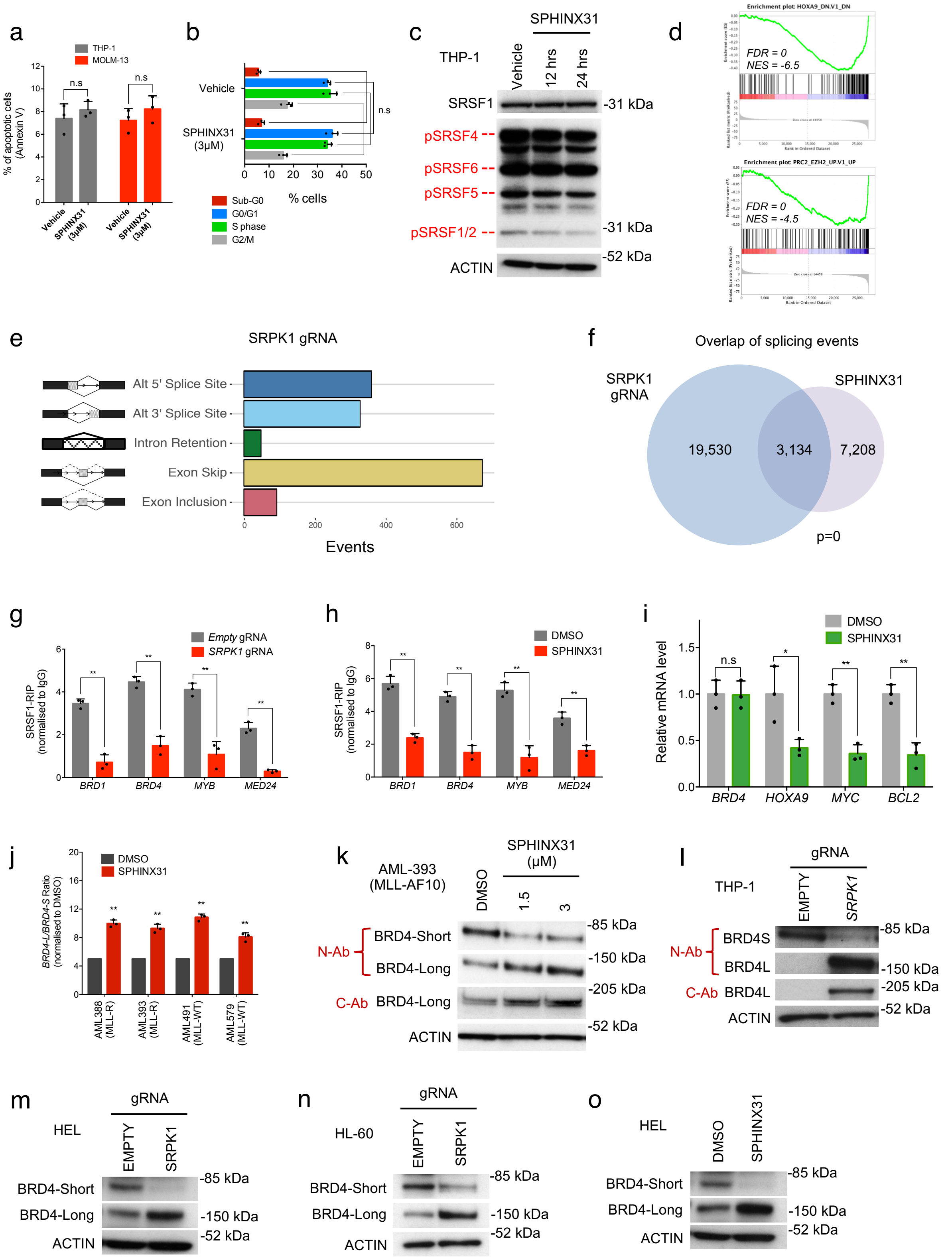

Supplementary Figure 8: Effects of SRPK1 inhibition of gene expression and splicing in AML cells

(a) Levels of apoptosis in THP-1 and MOLM-13 cells after 24 hours of treatment with 3µM SPHINX31 compared to vehicle (mean ± s.d., n=3). **(b)** Cell cycle analysis of THP-1 cells treated with 3µM SPHINX31 for 24 hours (mean ± s.d., n=3). **(c)** Western blots for total SRSF1 and panPhospho-SRSF (pSRSF) in THP-1 cell lysates after treatment with 3µM SPHINX31

for 12 and 24 hours. Bands corresponding to phosphorylated SRSF1, SRSF2, SRSF4, SRSF5 and SRSF6 are indicated. SRSF1 and SRSF2 proteins have the same size. **(d)** Examples of enriched oncogenic signature gene sets amongst genes significantly down-regulated upon treatment with 3µM SPHINX31 for 24 hours (logFC ≥ 1 and p_{adj} ≤ 0.05). For a full list of enriched oncogenic signatures see Supplementary Table 1). **(e)** Frequency and type of significantly altered splicing events (FDR ≤0.001) in THP-1 cells after gRNA targeting of *SRPK1*. **(f)** Overlap of spicing events after genetic or pharmacological inhibition of SRPK1 in THP-1 cells (hypergeometric test). **(g,h)** RIP-qPCR analyses using anti-SRSF1 antibody for selected targets in THP-1 cells after genetic **(g)** or pharmacological **(h)** inhibition of SRPK1 (mean ± s.d., n=3). **(i)** qRT-PCR quantitation after 72hr treatment of THP-1 cells with 1.5µM SPHINX31, showing significant decrease of *HOXA9*, *MYC* and *BCL2*, but not total *BRD4* mRNA levels (mean ± s.d., n=3). **(j)** qRT-PCR quantitation of the *BRD4L/BRD4S* ratio in patient derived AML samples after treatment with 1.5µM SPHINX31, illustrating a significant *BRD4S*-to-*BRD4L* isoform switch post-treatment with SPHINX31 irrespectve of AML subtype (mean ± s.d., n=3). **(k)** Western blot in human *MLL-X*-driven patient-derived cells after SPHINX31 treatment, showing a marked switch from the BRD4S to the BRD4L protein isoform using the same BRD4 antibodies as in main Figure 3. **(l)** Immunoblot in THP-1 cells on day 6 post-transduction with gRNA against *SRPK1*, showing a switch from BRD4S to BRD4L. **(m-o)** Western blot in human *MLL*-WT AML cell lines genetic **(m,n)** or pharmacological **(o)** inhibition of SRPK1, showing *BRD4S*-to-*BRD4L* switch using a BRD4 N-terminal antibody that detects both isoforms**.** n.s, not significant. * P<0.01 (t-test), ** P<0.001 (t-test).

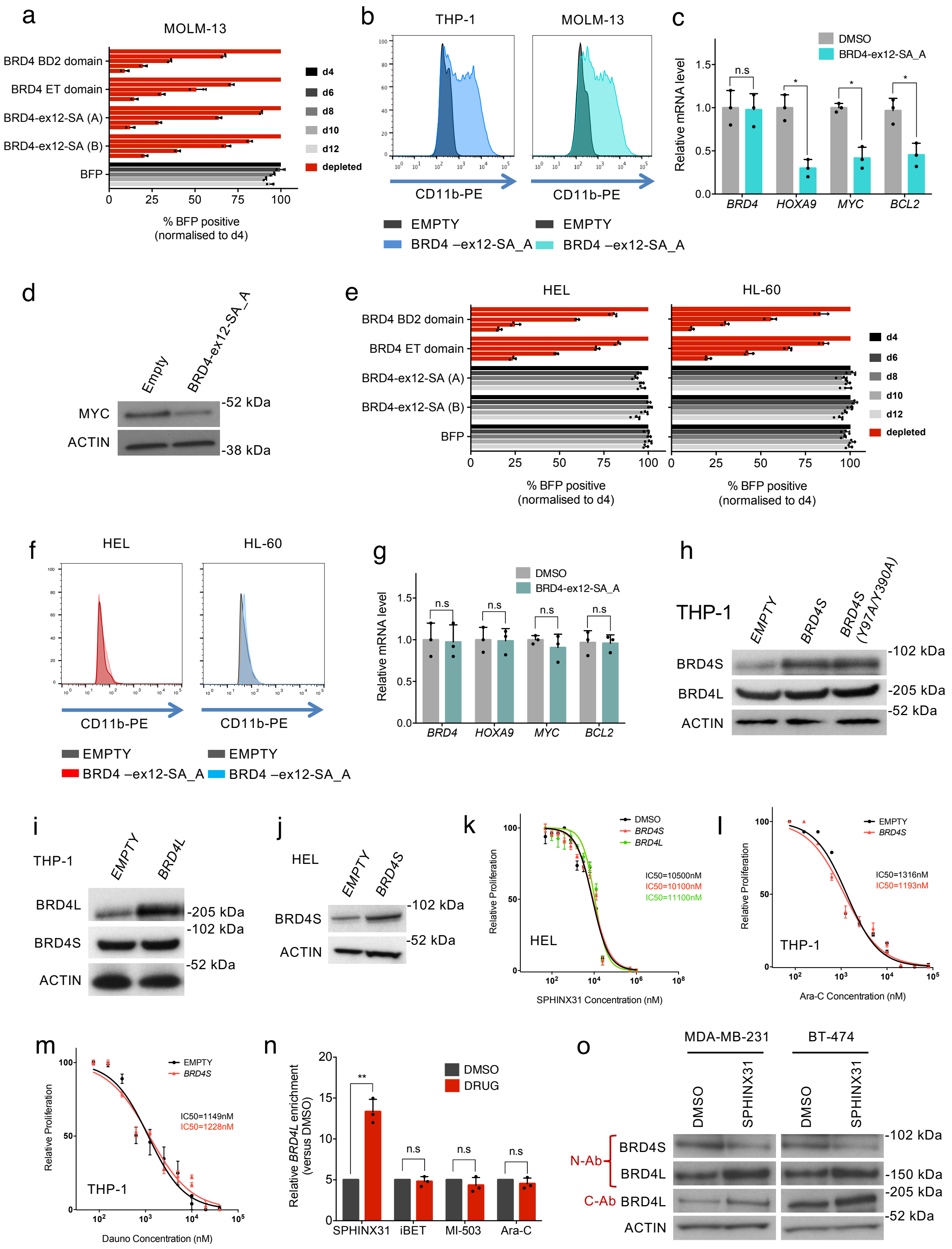

Supplementary Figure 9: Effects of the *BRD4S*-to-*BRD4L* isoform switching on gene expression in AML cells

(a) Competitive co-culture of MOLM-13 cells transfected with lentiviral gRNAs against *BRD4* (BFP positive) vs non-transfected cells normalized to %BFP on day 4 (mean ± s.d., n=3). gRNAs were designed against known essential *BRD4* domains (BD2 or ET) or the splice acceptor of the *BRD4S*-exon 12 (ex12-SA)**.** **(b)** Myeloid differentiation of THP-1 and MOML-13 cells targeted with empty gRNA or gRNA against BRD4-exon12 splice acceptor, 7 days post-transduction. **(c)** qRT-PCR results of THP-1 cells 7 days post-transduction with empty vector vs BRD4-ex12-SA gRNA, showing significant decrease of *HOXA9*, *MYC* and *BCL2* but not total *BRD4* mRNA (mean ± s.d., n=3). **(d)** Western blot of THP-1 cell lysates showing reduction of MYC protein upon *BRD4S*-to-*BRD4L* isoform switch using BRD4-ex12-SA-gRNA_A. **(e)** Competitive co-culture of HEL and HL-60 cells transfected with lentiviral gRNAs against *BRD4* (BFP positive) vs non-transfected cells normalized to %BFP on day 4 (mean ± s.d., n=3). gRNAs were designed against known essential *BRD4* domains (BD2 or ET) or the splice acceptor of the *BRD4S*-exon 12**.** **(f)** CD11b expression in HEL and HL-60 cells targeted with empty gRNA or gRNA against BRD4-exon12 splice acceptor, 7 days post-transduction**,** showing no effect on myeloid differentiation. **(g)** qRT-PCR results of HEL cells 7 days post-transduction with empty vector vs gRNA against the BRD4-exon12 splice acceptor, showing no significant effect on the expression of *HOXA9*, *MYC*, *BCL2* or *BRD4* mRNA (mean ± s.d., n=3). Student's t-test was performed. **(h,i)** Western blot of THP-1 cell lysates for BRD4S, BRD4L and ACTIN demonstrating increased protein levels of BRD4S **(h)** or BRD4L **(i)** after ectopic expression of wild-type and double bromodomain mutant (Y97A/Y390A*)* *BRD4S* or wild-type *BRD4L*. **(j)** Western blot of HEL cell lysates showing increased protein levels of BRD4S after ectopic expression of wild-type *BRD4S*. **(k)** Dose-response curves of HEL cells to SPHINX31 after overexpression of wild-type *BRD4S* or *BRD4L*, showing no effect on the SPHINX31 IC50. **(l,m)** Dose-response curves of THP-1 cells to cytarabine (Ara-C) **(l)** or daunorubicin (Dauno) **(m)** after overexpression of wild-type *BRD4S*, showing no significant change on the IC50 of either drugs. **(n)** qRT-PCR for *BRD4L* after 72 hours treatment of THP-1 with IC50 for SPHINX31 (1.5μM), iBET-151 (400nM), MI-503 (1μM) and Ara-C (80nM), showing significant enrichment of *BRD4L* exclusively with SPHINX31 treatment (mean ± s.d., n=3). **(o)** Western blot of lysates from breast cancer cell lines MDA-MB-231 and BT-474 after 48hrs treatment with 1.5μM SPHINX31, showing reduction of BRD4S/BRD4L protein isoform ratio (same antibodies as main Figure 3). n.s, not significant. * P<0.01 (t-test), ** P<0.001 (t-test).

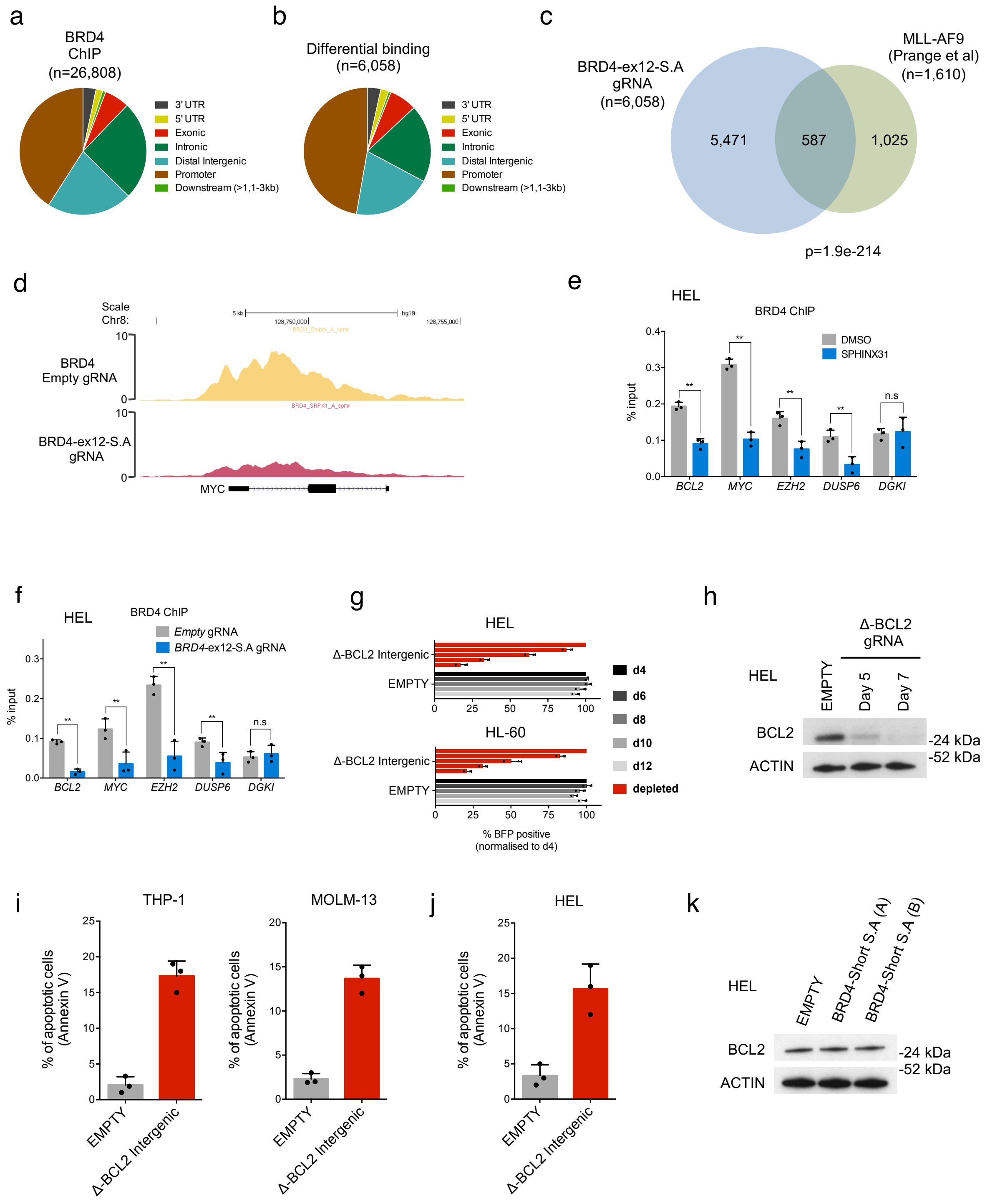

Supplementary Figure 10: Disruption of BRD4 splicing has no effect on BCL2 expression in *MLL-WT* AML cells

(a,b) Pie charts of genomic regions bound by BRD4 (a) and those displaying reduced binding upon *BRD4S*-to-*BRD4L* switch (b) as determined by ChIP-seq. **(c)** Overlap between MLL-AF9 specific loci from Prange et al[23] and loci with reduced BRD4 binding after *BRD4S*-to-*BRD4L* isoform switching induced by BRD4-ex12-SA_A gRNA. THP-1 cells were used for both ChIPseq datasets. **(d)** BRD4 ChIP-seq of THP-1 cells targeted with empty gRNA or gRNA BRD4-ex12-SA_A, 5 days post-transduction, showing significant eviction of BRD4 from the *MYC* promoter with the latter. **(e,f)** ChIP-qPCR experiments in HEL cells showing reduced

binding of BRD4 after exposure to 1.5µM SPHINX31 or BRD4-ex12-SA_A gRNA (6 days post-transduction). (mean ± s.d., n=3). **(g)** Competitive co-culture showing the requirement for the *BCL2* 3' enhancer for HEL and HL-60 cell growth and proliferation. Results were normalized to day 4 (mean ± s.d., n=3). **(h)** Reduction of BCL2 protein in HEL cells by gRNA targeting of the 3' BCL2 enhancer **(i,j)** Apoptosis levels in THP-1, MOLM-13 and HEL cells targeted with dual gRNA against the *BCL2* 3' enhancer. Results normalized to empty vector (mean ± s.d., n=3). **(k)** Western blot for BCL2 in HEL cells transduced with BRD4-ex12-SA_A gRNA, displaying no effect on total BCL2 protein levels upon *BRD4S*-to-*BRD4L* isoform switch. ** P<0.001 (t-test).

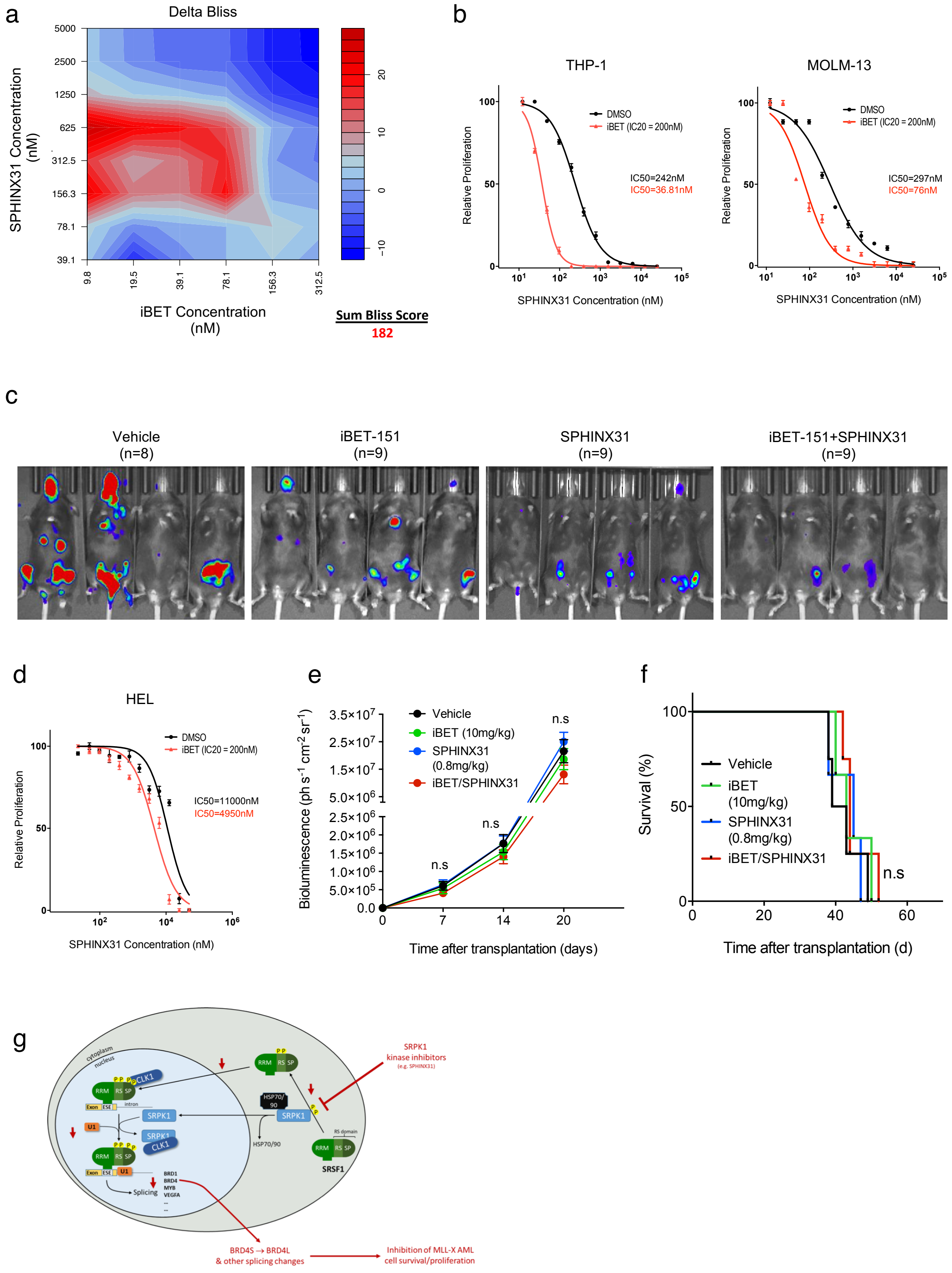

Supplementary Figure 11: Synergy between SPHINX31 and iBET-151 in *MLL-X* AML cells

(a) Bliss drug synergy plot and excess over Bliss sum for the indicating dose combinations between SPHINX31 and iBET-151. Synergy is indicated with all shades of red (scale bar). **(b)** Increased growth inhibition of THP-1 and MOLM-13 cells by SPHINX31, in the presence of 200nM (IC20) iBET-151 (mean ± s.d., n=3). **(c)** Bioluminescence imaging of mice related to

figures 4j-k. **(d)** Effect of growth in HEL cells by SPHINX31, in the presence of 200nM (IC20) iBET-151 (mean ± s.d., n=3). **(e)** Quantification of luminescence for the mice transplanted with luciferase-labeled HEL cells and treated with low dose of iBET-151 (10mg/kg) or SPHINX31 (0.8mg/kg) or both, showing no synergistic effect between the two drugs. **(f)** Survival of HEL transplanted mice treated as described in **(e)**. n=4 animals per group. Log-rank (Mantel-Cox) test was performed with no significant difference between any comparison of treatment-groups. **(g)** Schematic model of the mode of action for SRPK1 inhibition in MLL-fusion leukaemia. SRPK1 phosphorylates serine-arginine (SR) splicing factors such as SRSF1 at the RS-rich part of their SR domain to enable their nuclear entry. Once in the nucleus, they get further phosphorylated at the serine-proline (SP)-rich part of their SR domain by CLK1 and this enables them to bind at exonic splice enhancers (ESE) of nascent mRNAs. With successive phosphorylation events, the affinity of CLK1 to SRSF1 is thought to be reduced such that SRPK1 can bind and remove it from the later, an event that is not thought to rely on SRPK1's kinase function. At that stage SRSF1 can recruit the U1 small nuclear ribonucleoprotein and splicing begins. Inhibition of SRSF1 phosphorylation by SRPK1 reduces SRSF1 nuclear entry and this in turn inhibits the downstream events (red arrows) and alters splicing of several genes including BRD4 and others required for the survival and proliferation of AMLs driven by common *MLL-X* fusion genes. n.s, not significant.

Full uncropped Western blot scans

Figure 1g

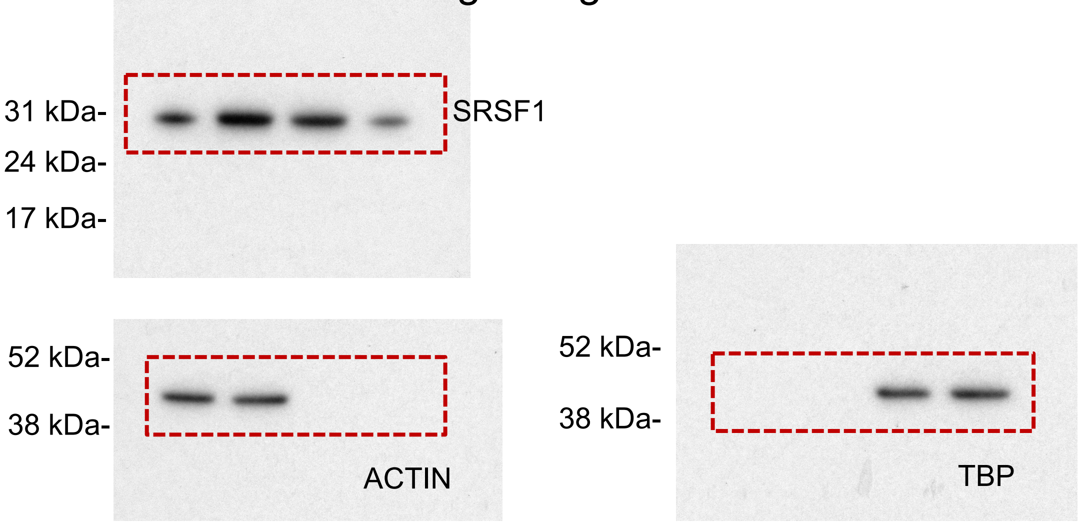

Supplementary Figure 1a

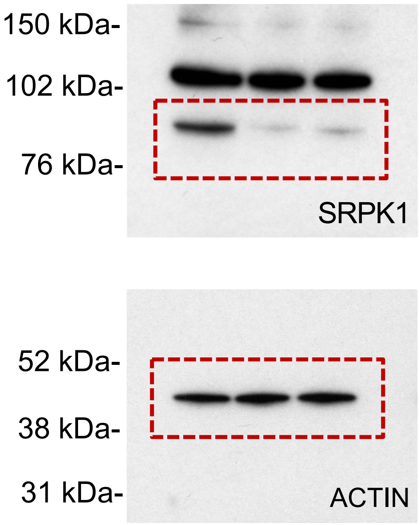

Supplementary Figure 1b

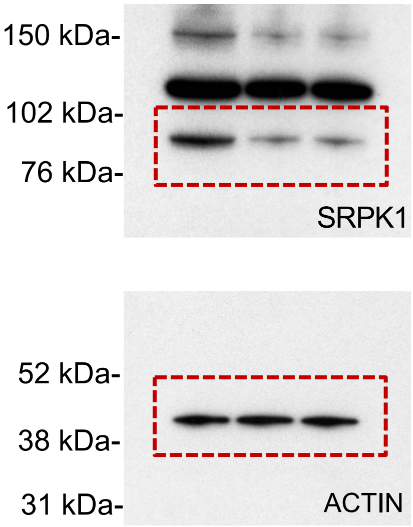

Supplementary Figure 1c

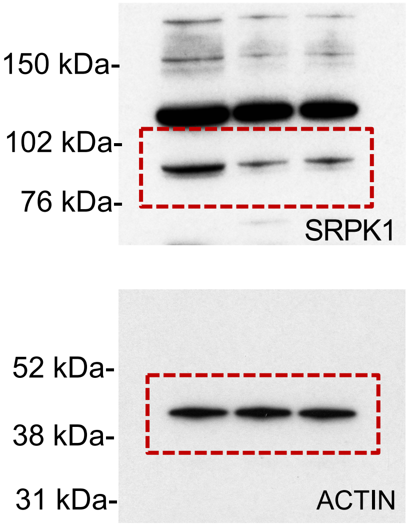

Supplementary Figure 1d

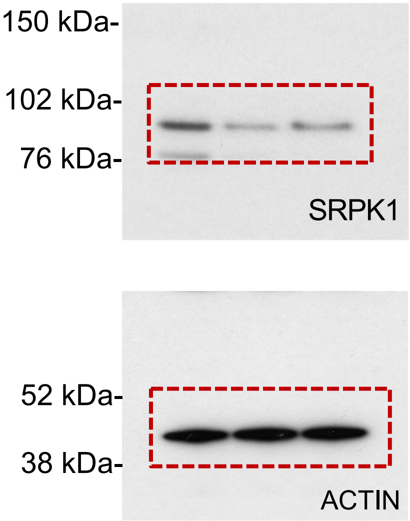

Supplementary Figure 1e

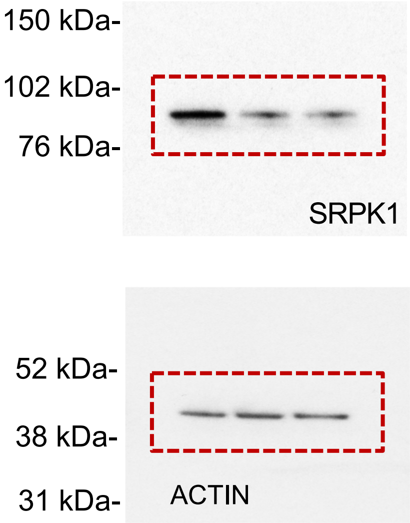

Supplementary Figure 1f

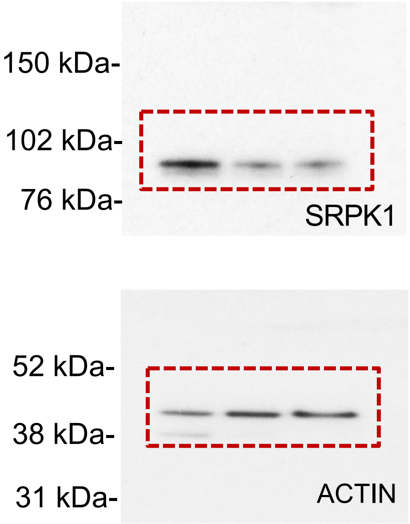

Supplementary Figure 2e

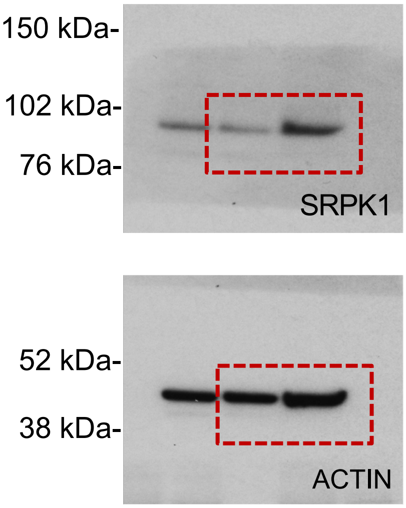

Supplementary Figure 3a

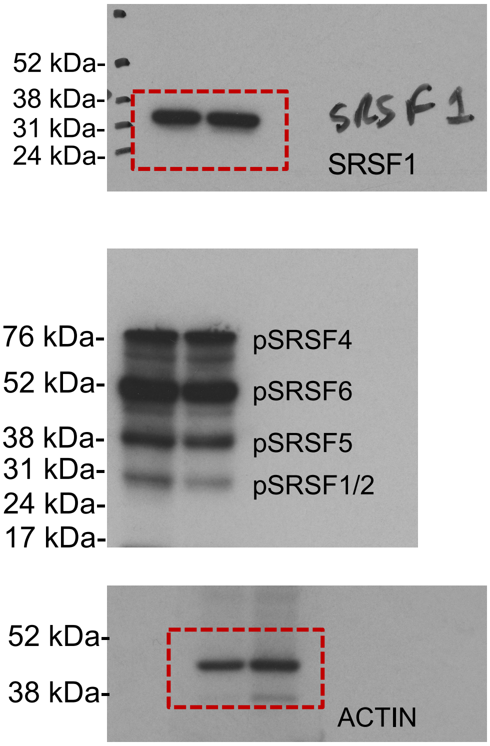

Supplementary Figure 3b

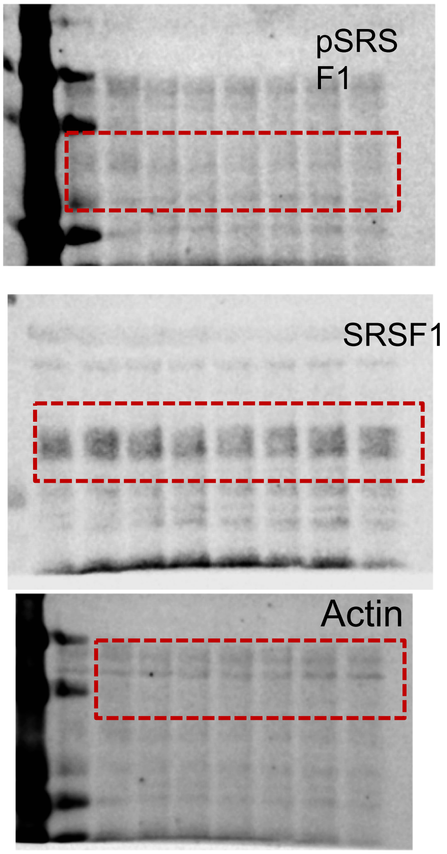

Supplementary Figure 3g

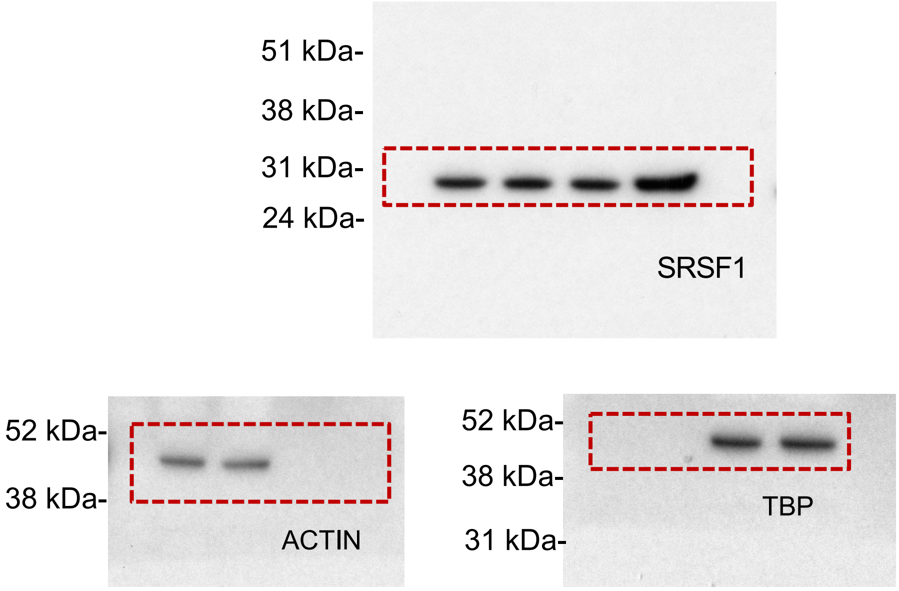

Supplementary Figure 3h

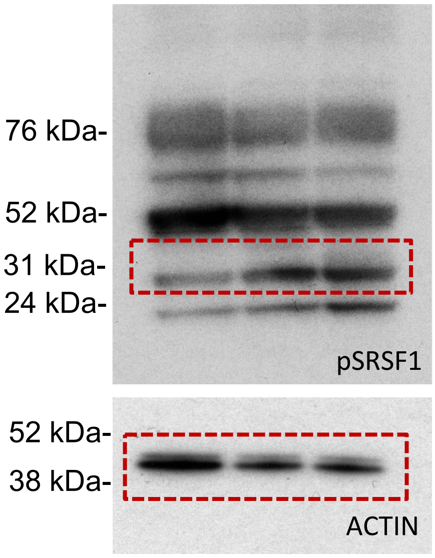

Supplementary Figure 3i

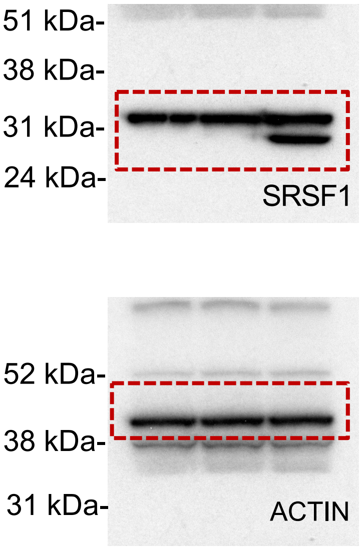

Supplementary Figure 6c

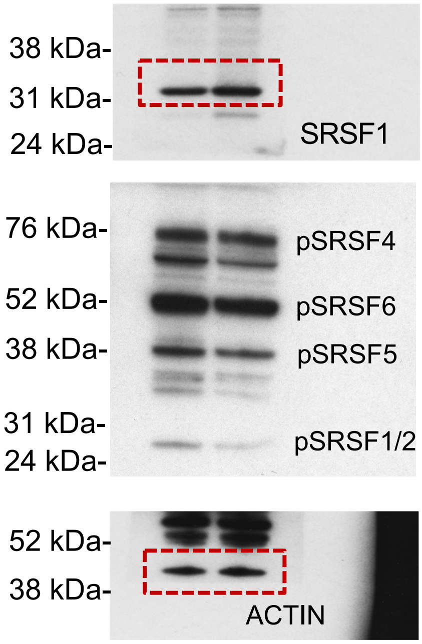

Supplementary Figure 6g

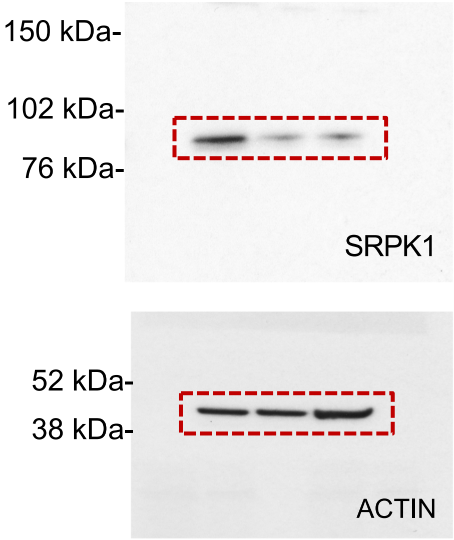

Figure 3g

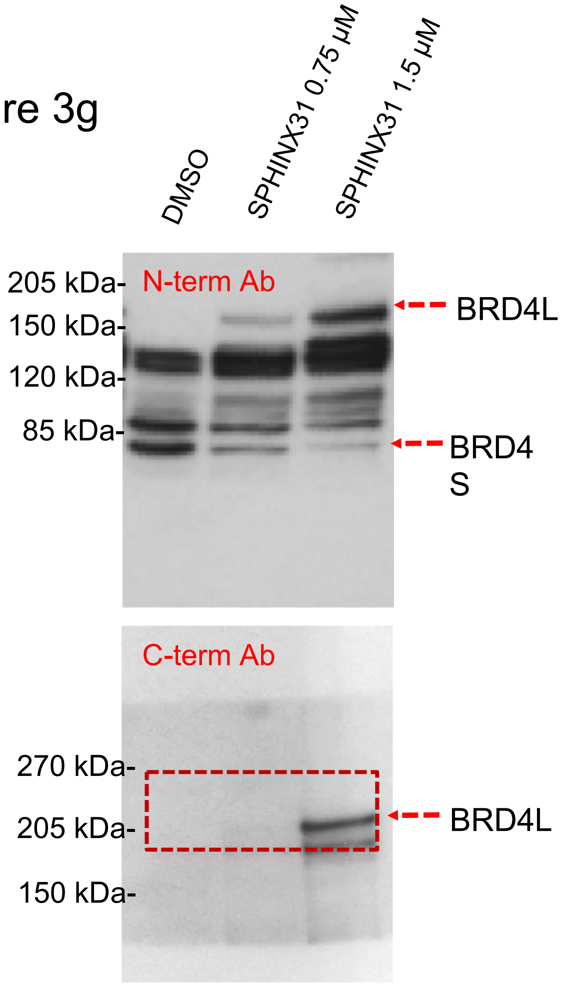

Figure 3i

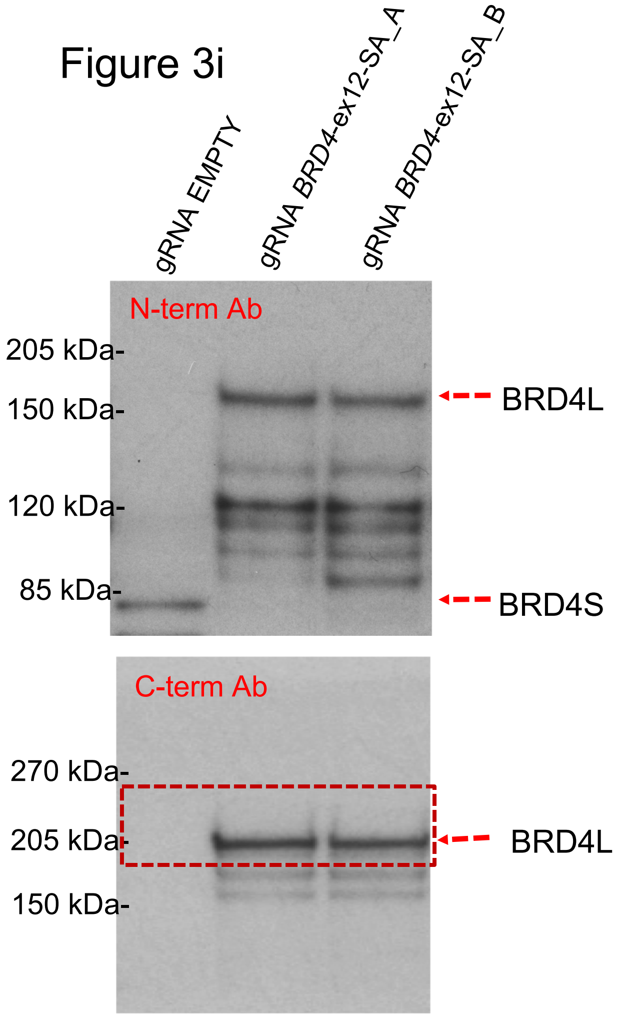

Figure 3g

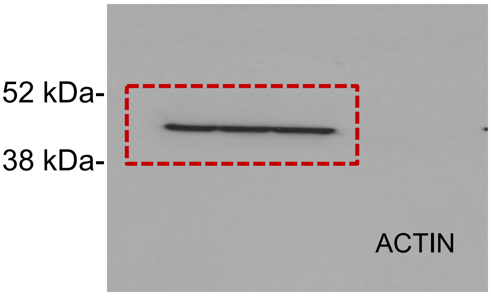

Figure 3i

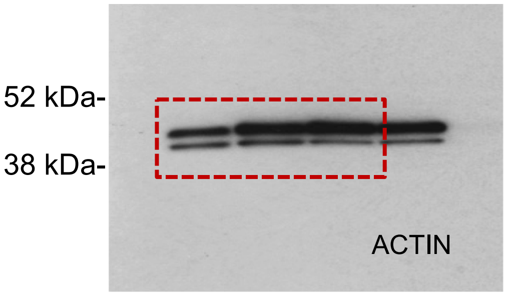

Figure 3l

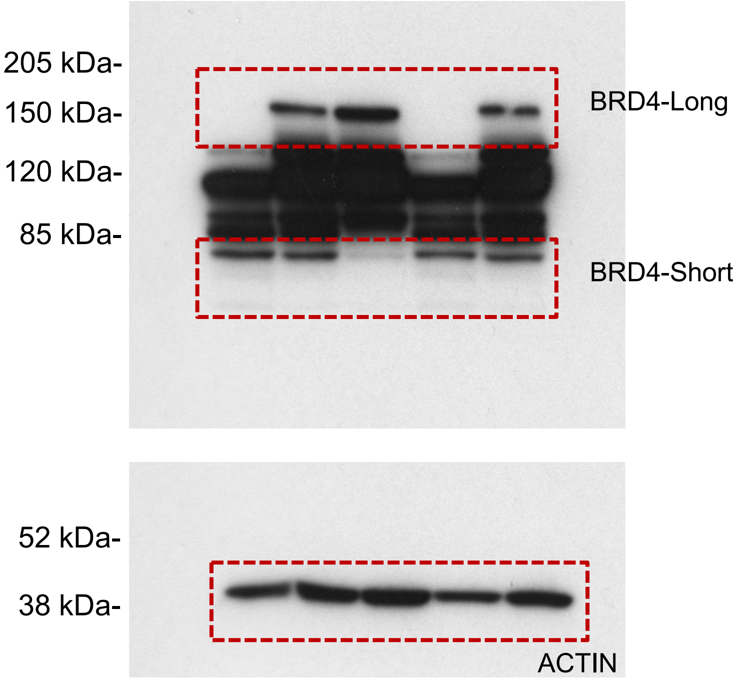

Supplementary Figure 7c

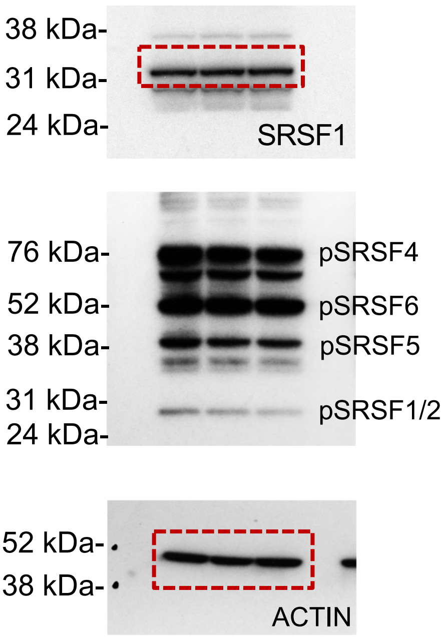

Supplementary Figure 7k

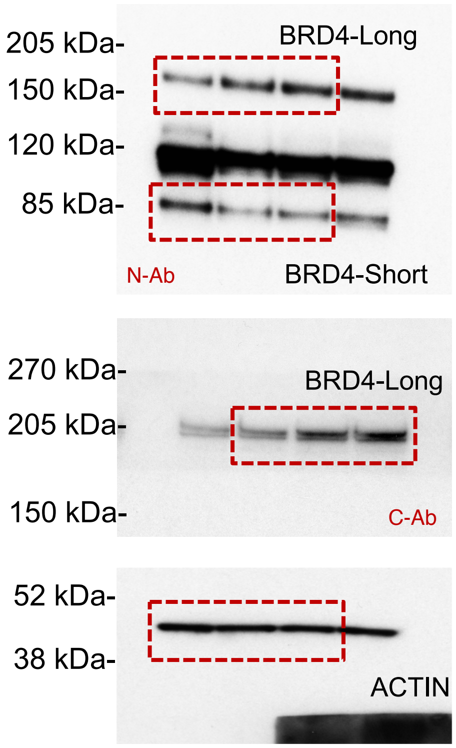

Supplementary Figure 7i

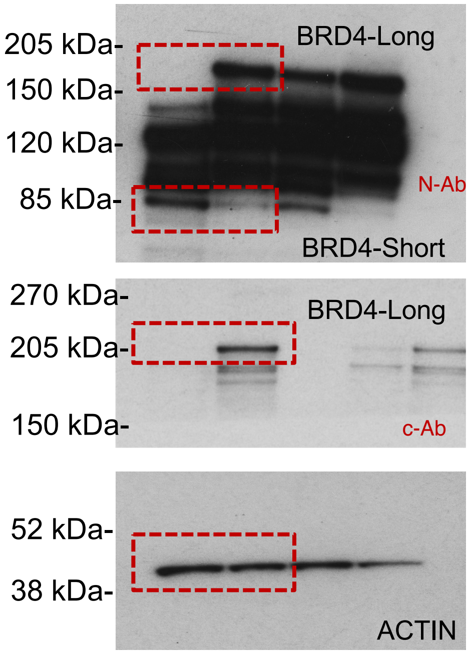

Supplementary Figure 7m

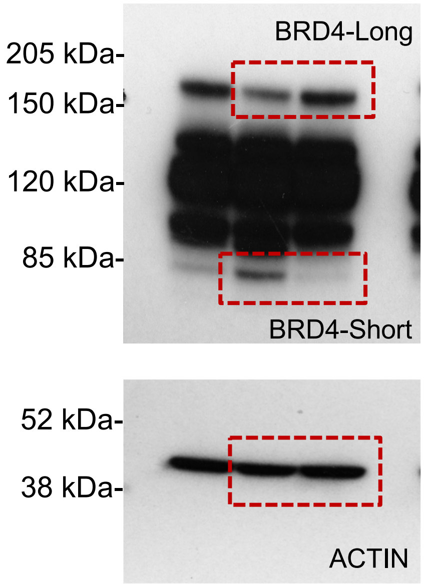

Supplementary Figure 7n

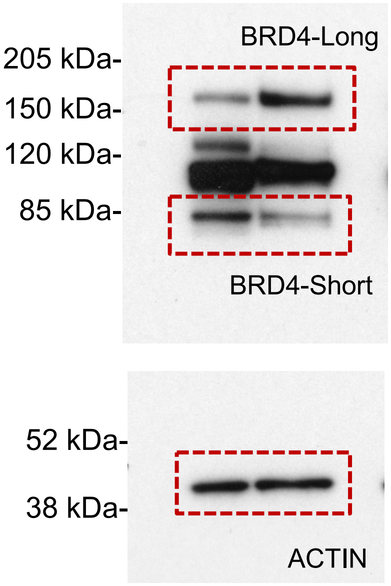

Supplementary Figure 7o

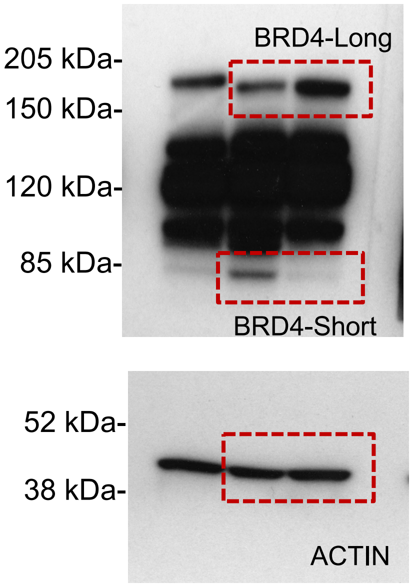

Supplementary Figure 8d

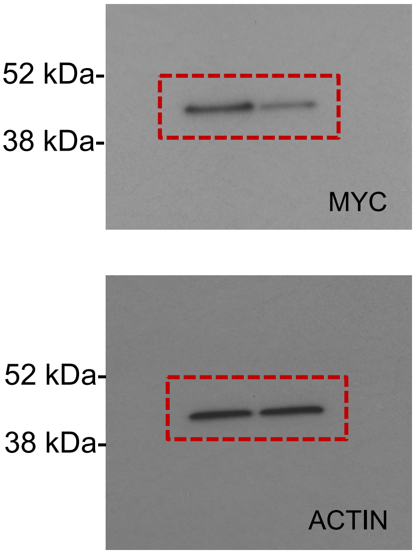

Supplementary Figure 8h

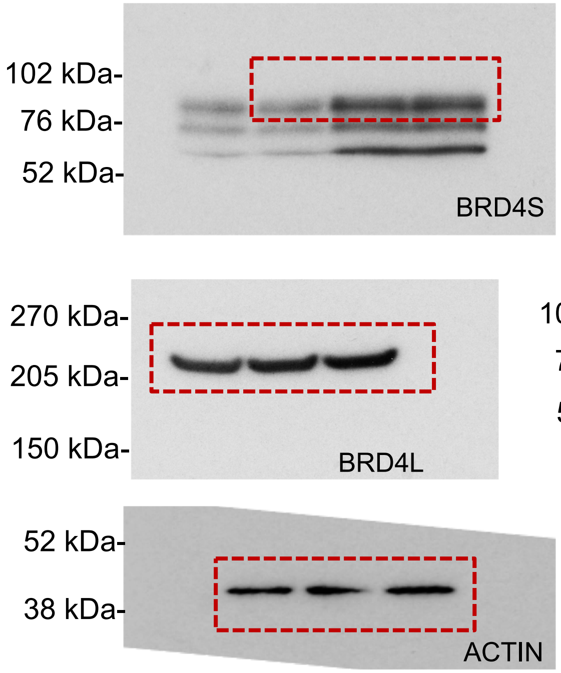

Supplementary Figure 8i

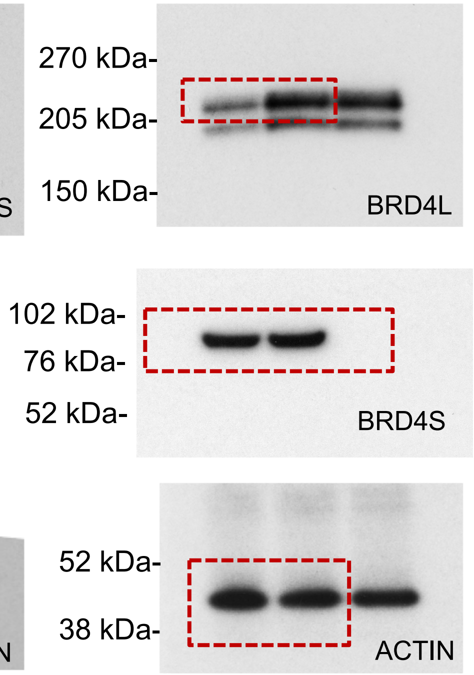

Supplementary Figure 8j

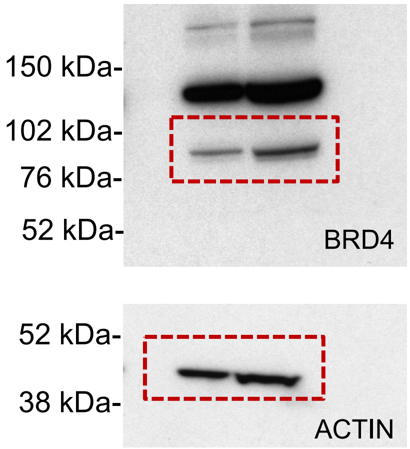

Supplementary Figure 8o

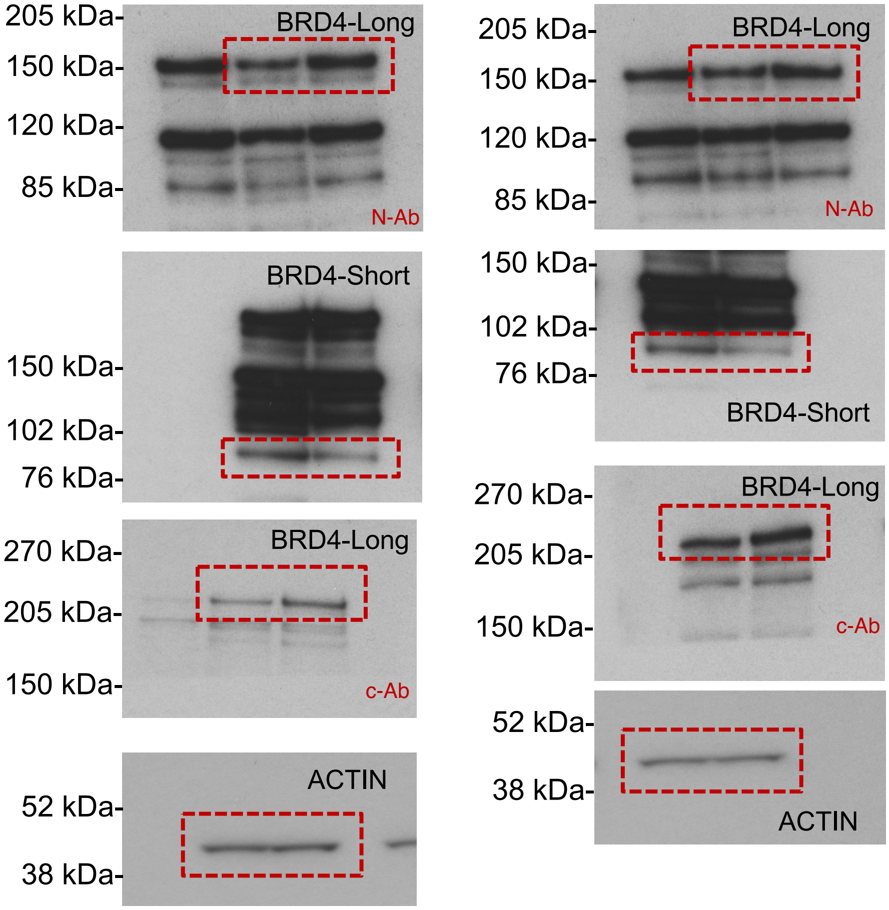

Figure 4h

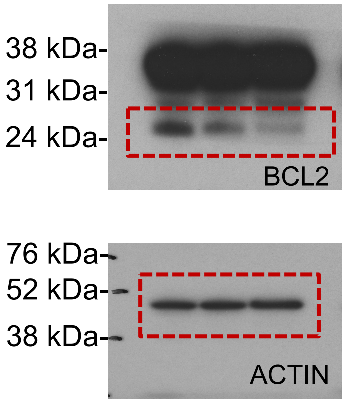

Figure 4i

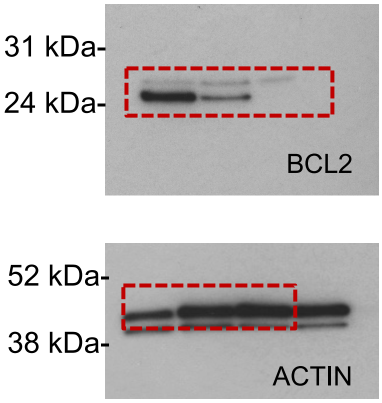

Supplementary Figure 9h

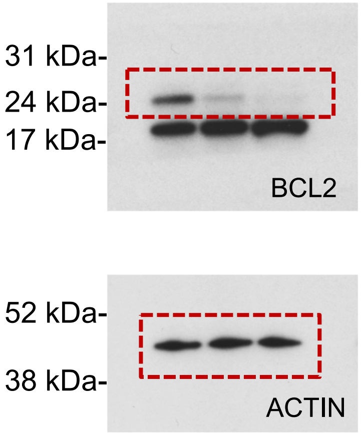

Supplementary Figure 9k

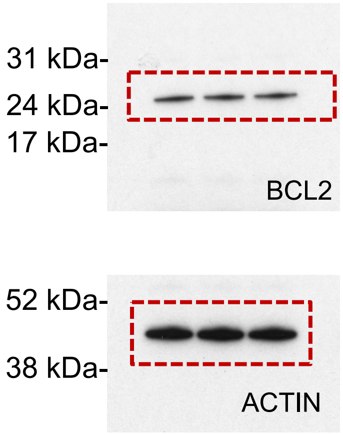

Gating Strategy for FACS experiments

Example of gating strategy employed for flow cytometry experiments (Related to Figures 1, 3, 4 and Supplementary Figures 2, 8 and 9)

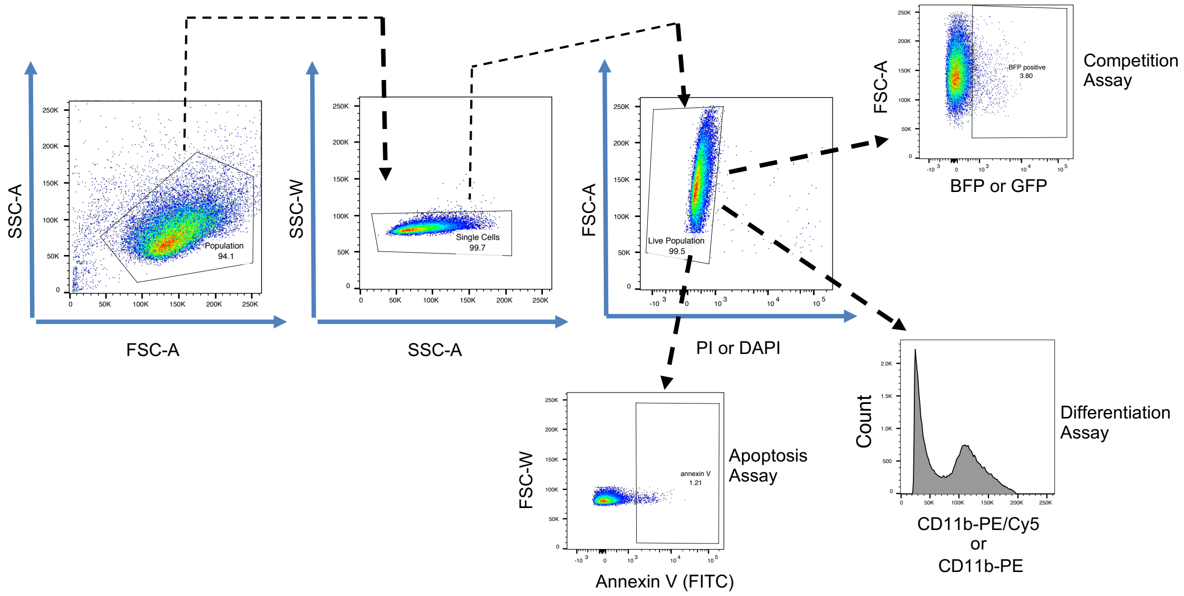

Gating strategy and FMO controls for the determination of the HSPC levels upon treatment with Vehicle or SPHINX31 (Related to Figure 2 and Supplementary Figure 6)

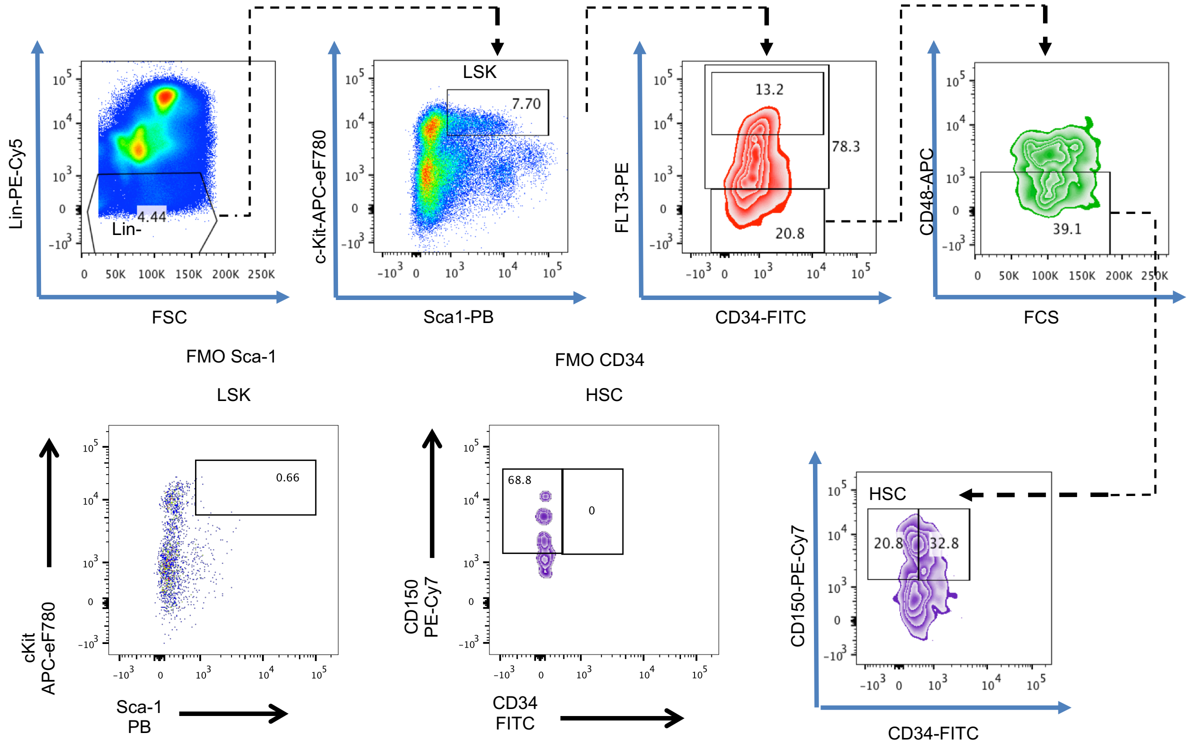

Examples of splicing modulation of selected genes upon pharmacological inhibition of SRPK1

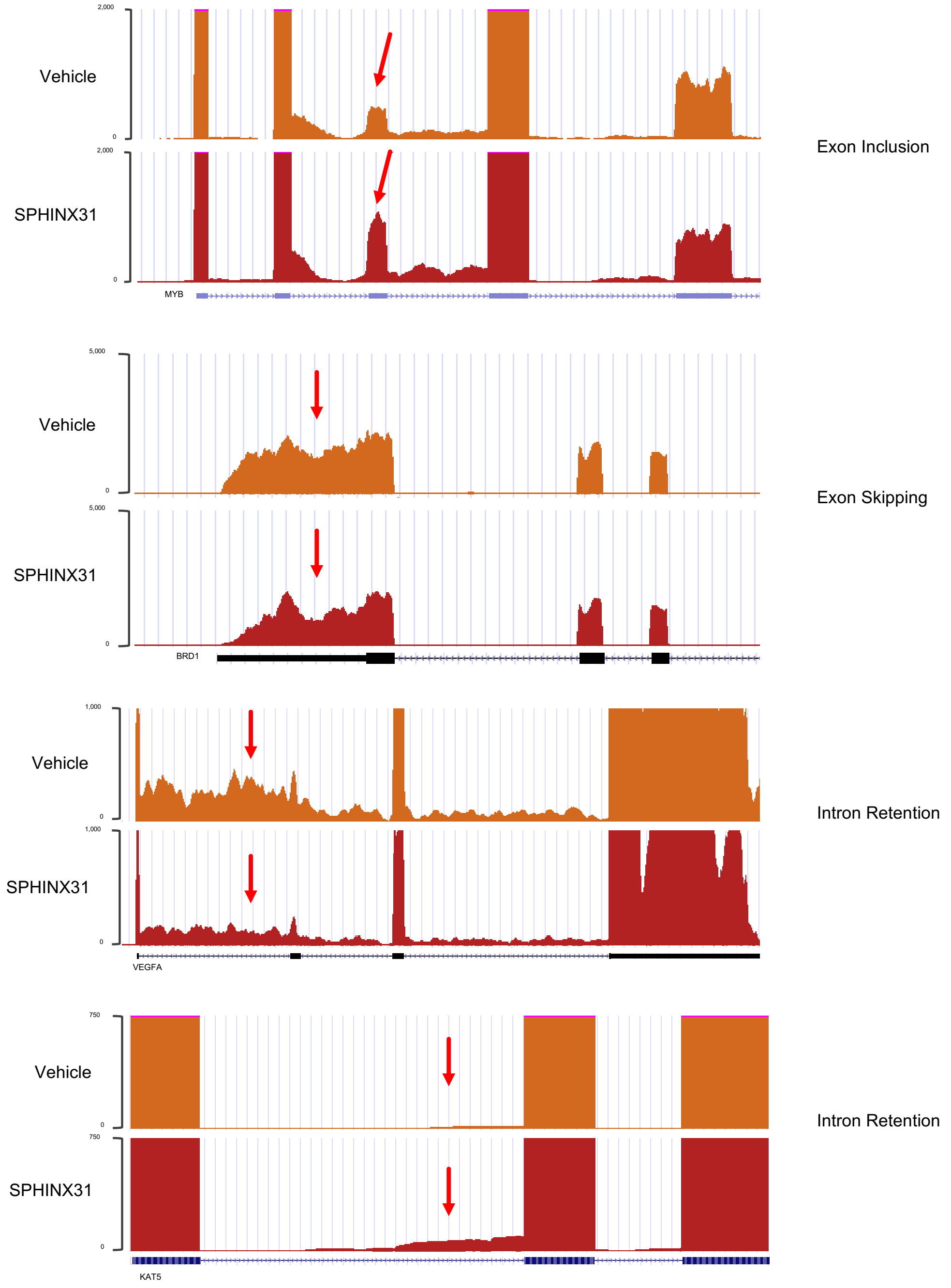

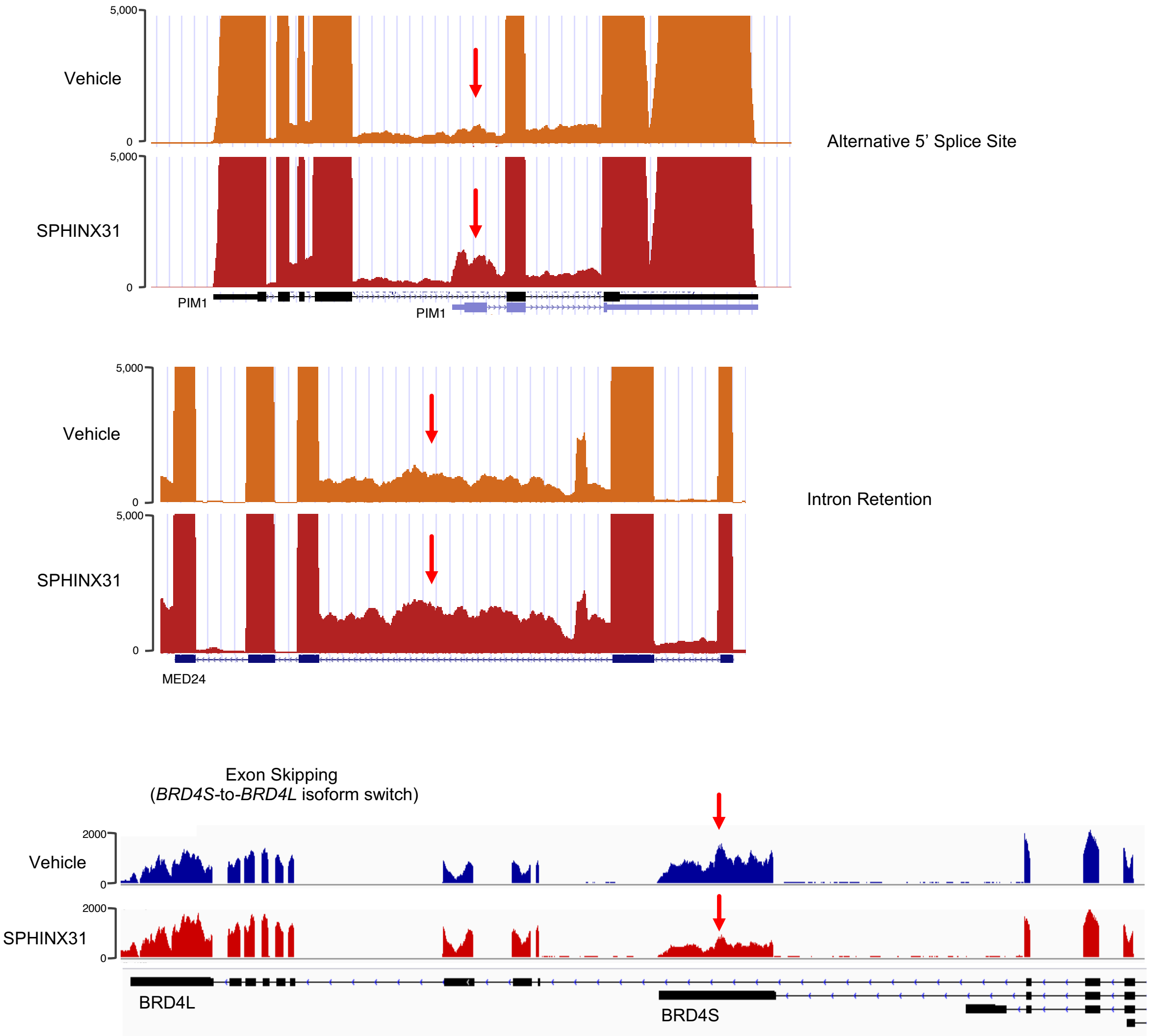

BRD4S-to-*BRD4L* isoform switch upon pharmacological inhibition of SRPK1
